# Neuroanatomical and neurophysiological mechanisms of acoustic and weakly electric signaling in synodontid catfish

**DOI:** 10.1002/cne.24920

**Published:** 2020-04-21

**Authors:** Loïc Kéver, Andrew H. Bass, Eric Parmentier, Boris P. Chagnaud

**Affiliations:** ^1^ Laboratoire de Morphologie Fonctionnelle et Evolutive Université de Liège Liège Belgium; ^2^ Department of Neurobiology and Behavior Cornell University Ithaca New York USA; ^3^ Department Biology II Ludwig‐Maximilians‐University Munich Planegg Germany; ^4^ Institute for Biology Karl‐Franzens‐University Graz Graz Austria

**Keywords:** AB_2337244, AB_2337249, animal vocalization, catfishes, electric fish, motor neurons, patch‐clamp technique, premotor neurons

## Abstract

To what extent do modifications in the nervous system and peripheral effectors contribute to novel behaviors? Using a combination of morphometric analysis, neuroanatomical tract‐tracing, and intracellular neuronal recording, we address this question in a sound‐producing and a weakly electric species of synodontid catfish, *Synodontis grandiops*, and *Synodontis nigriventris*, respectively. The same peripheral mechanism, a bilateral pair of protractor muscles associated with vertebral processes (elastic spring mechanism), is involved in both signaling systems. Although there were dramatic species differences in several morphometric measures, electromyograms provided strong evidence that simultaneous activation of paired protractor muscles accounts for an individual sound and electric discharge pulse. While the general architecture of the neural network and the intrinsic properties of the motoneuron population driving each target was largely similar, differences could contribute to species‐specific patterns in electromyograms and the associated pulse repetition rate of sounds and electric discharges. Together, the results suggest that adaptive changes in both peripheral and central characters underlie the transition from an ancestral sound to a derived electric discharge producing system, and thus the evolution of a novel communication channel among synodontid catfish. Similarities with characters in other sonic and weakly electric teleost fish provide a striking example of convergent evolution in functional adaptations underlying the evolution of the two signaling systems among distantly related taxa.

## Graphical abstract

Synodontid catfishes use an Elastic Spring Apparatus, including protractor muscles, to produce electric discharges or sounds. Anatomical characterization, together with motoneuron patch‐clamp recordings and protractor muscle EMGs suggest that adaptive changes in peripheral and central characters underlie the transition from an ancestral sound to a derived electric discharge producing system.

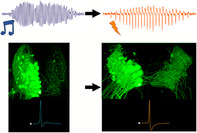

**Abbreviations**
ADP: afterdepolarization
AHP: afterhyperpolarization
AP: action potential
ED: electric discharge
EMG Ref: recording from EMG reference electrode
EMG: electromyogram
ESA: elastic spring apparatus
MN: protractor motoneurons
MR: Müllerian ramus
NC: neurocranium
NS: nuchal shield
P: plate of the Müllerian ramus
PM R: EMG recording from right protractor muscle
PM L: EMG recording from left protractor muscle
PM: protractor muscle
PN: protractor nucleus
PN1: Type 1 premotor neurons
PN2: Type 2 premotor neurons
PN3: Type 3 premotor neurons
Pr: process of the Müllerian ramus
*S.g*.: *Synodontis grandiops*
*S.n*.: *Synodontis nigriventris*
SA: sarcoplasm
SL: standard length
SNR: signal‐to‐noise ratio

## 1 INTRODUCTION

A central goal of comparative and evolutionary neurobiology is to determine the extent to which modifications in neural circuits parallel differences in behavior among closely related species (Katz, 2016; Katz & Harris‐Warrick, 1999). Changing the connectivity pattern of interneurons in spinal circuits, for instance, results in a change from an alternating to a simultaneous (i.e. hopping) gait (Kiehn, 2016). Similarly, alterations of synaptic strength and intrinsic membrane properties in a three‐neuron stomatogastric network can result in the generation of gastric mill patterns with similar or different periods and burst durations (Prinz, Bucher, & Marder, 2004). Temporal differences in the courtship calls of pipid frogs (*Xenopus*) can be explained, in part, by the intrinsic properties of premotor neurons (Barkan, Kelley, & Zornik, 2018), while divergent spectral features appear to be intrinsic to the larynx (Kwong‐Brown et al., 2019). While these studies demonstrate that species diversity in an acoustic signal can depend on both central and peripheral properties within a single genus, it remains an open question as to what might accompany the evolution of novel behaviors. One strategy to best identify such characters would be to compare closely related species having the same muscle accomplishing two different functions. The protractor motor system of synodontid catfish offers such an opportunity because it is used for the production of sound, weak electric fields, or both, depending on the species (Baron, Morshnev, Olshansky, & Orlov, 1994; Boyle, Colleye, & Parmentier, 2014; Hagedorn, Womble, & Finger, 1990; Orlov & Baron, 2005; Orlov, Baron, & Golubtsov, 2017).

The protractor muscle of synodontids inserts on the Müllerian ramus, a vertebral process that ends in a plate‐like structure lying on the swim bladder wall; together referred to as an elastic spring apparatus (ESA) (Parmentier & Diogo, 2006). Comparative studies strongly suggest that the ancestral function of the ESA is sound production (Boyle et al., 2014). Although electrogenic synodontids retain an ESA, their protractor muscle fibers have many fewer myofibrils (Boyle et al., 2014), like the myogenic electric organ of other genera of weakly electric fish (e.g., Bass, Denizot, & Marchaterre, 1986; Bennett, 1971). The protractor muscles are innervated ipsilaterally by a protractor motor nucleus containing dense clusters of motoneurons that extends along the midline from the caudal hindbrain into the rostral spinal cord (Hagedorn et al., 1990; Ladich & Bass, 1996). Populations of protractor premotor neurons are located in the dorsolateral part of the motor nucleus (Type I) and close to the rostral end of the motor nucleus, lateral to the medial longitudinal fasciculus (Type 2) (Ladich & Bass, 1996). Ladich and Bass (1996) also report extensive connections between this premotor‐motor circuit and a region comparable in location to the vocal prepacemaker nucleus in the toadfish vocal system (Bass, Marchaterre, & Baker, 1994; Chagnaud, Baker, & Bass, 2011; Chagnaud & Bass, 2014), suggesting a hindbrain vocal network comparable in organization to that of toadfishes.

**FIGURE 1:**
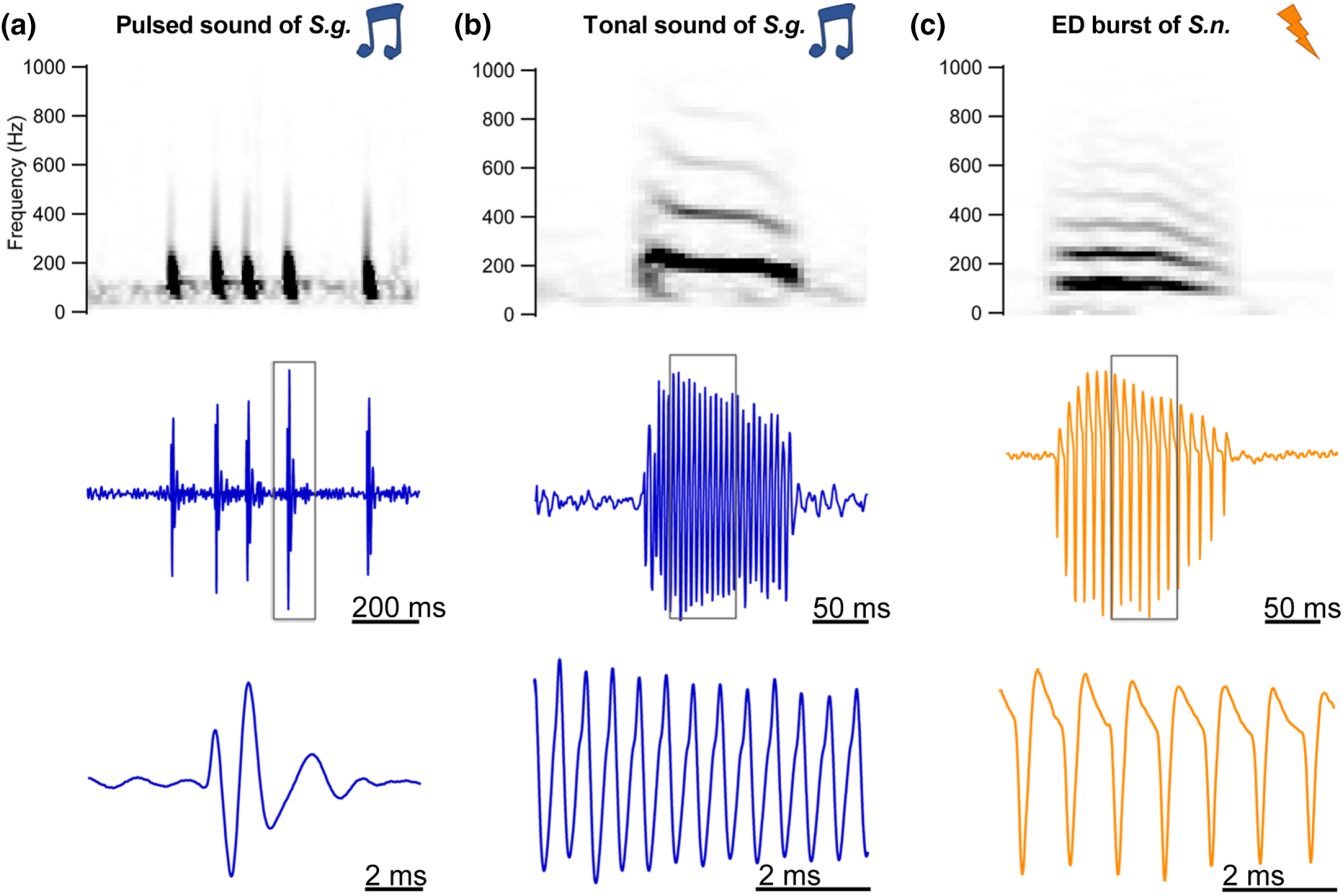
Swim bladder sounds and electric discharges (EDs) recorded from *Synodontis grandiops* and *S. nigriventris*, respectively. (a) Spectrogram (top) and waveform (middle and bottom at two timescales) of a pulse train produced by a *S. grandiops* (*S.g*.). (b) Spectrogram (top) and waveform (middle and bottom at two timescales) of a tonal sound produced by a *S. grandiops* (*S.g*.). (c) Spectrogram (top) and waveform (middle and bottom at two timescales) of an ED burst produced by a *S. nigriventris* (*S.n*.). Water temperature: 26 ± 1°C [Color figure can be viewed at wileyonlinelibrary.com]

To uncover adaptive features associated with the production of either sound or electric discharge (ED), we investigated neuroanatomical and neurophysiological characters of the ESA communication system in two species that show only one type of signaling mechanism; the sound‐producer *Synodontis grandiops* and the ED*‐*producer *Synodontis nigriventris* (Figure 1). We investigated the signal output (sound or ED) and morphology of the ESA, and recorded electromyograms (EMGs) of the protractor muscles that lead to such different signals. To understand how protractor muscle activity might be controlled, the hindbrain network, as well as the intrinsic membrane properties of protractor motoneurons, were studied. We found that the general organization of the ESA and its associated premotor‐motor network was similar in both sonic and ED species; however, multiple ESA and premotor‐motor morphological characters differed. Neurophysiological measures further indicated that the motoneuron populations in both species control fast‐synchronous activation of the paired protractor muscles; however, there were species‐specific patterns that could account for differences in sound and electric discharge pulse repetition rate.

## 2 MATERIAL AND METHODS

### 2.1 Animals

Twenty‐five *S. grandiops* (standard length, SL: 54 to 108 mm) purchased from “Les aquariums de Marbais” (Belgium) and 33 *S. nigriventris* (SL: 42 to 71 mm) purchased from “EFS Nürnberg” (Germany) were maintained at either Liège University or the Ludwig‐Maximilians‐University (LMU) in monospecific tanks of ~200 L (photoperiod: 12:12 hr L:D; water temperature: 26 ± 1°C). The tanks were supplied with numerous hiding places and the fish were fed daily with commercial fish food. While not all animals were sexed, observations of males and females did not reveal any obvious sexual dimorphism in the ESA. As the animals were acquired from the aquarium trade, their age was undetermined. All experimental procedures were approved by the Institutional Animal Care and Use Committee of the University of Liège (protocols 1,970 and 2,110) and the Regierung von Oberbayern (55.2‐1‐54‐2532‐13‐2016).

### 2.2 Sound analysis

Fish from both species were recorded following the protocol described in Boyle et al. (2014). During each trial, the simultaneous recording of sounds and EDs were performed. Synodontids also generate high‐frequency stridulation sounds by rubbing their pectoral spine against their pectoral girdle (Parmentier et al., 2010). These sounds were produced by both species, but were not analyzed as the focus here is on the shared motor system activating swim bladder‐associated muscles that are either sonic or electrogenic. Sounds were recorded with an HTI‐Min 96 hydrophone (−186.4 dBV re 1 μPa, frequency response 2 Hz–30 kHz; High Tech Inc., Long Beach, MS) or an AS‐1 hydrophone (−208dBV re 1 μPa, frequency response 1 Hz to 100 kHz; Aquarian Audio, Anacortes, WA) with a PA‐4 hydrophone preamplifier (Aquarian Audio), while EDs were recorded with two Teflon‐coated silver electrodes (4 cm exposed tips) separated by 25 cm. These electrodes were connected to a differential amplifier (A‐M Systems Model 1,700) that filtered (bandpass: 10 Hz–10 kHz; notch filter: on) and amplified the signal (10 k fold). The hydrophone and the amplifier were connected to an external sound card (Creative model SB0270; Creative Labs, Singapore or UltraLite‐mk3; MOTU; Cambridge, MA), and the signals were recorded on a laptop using Adobe Audition 2.0 (Adobe, San Jose, CA) or audacity 2.0.5 (http://sourceforge.net/projects/audacity/).

Swim bladder sounds were recorded from seven *S. grandiops* in two different glass tanks (tank 1: 60 × 29 cm, water depth kept at ~20 cm; tank 2: 108 × 48 cm, water depth kept at ~30 cm) and the EDs from four *S. nigriventris* in a plastic tank (45 × 27 cm, water depth kept at ~20 cm). For both types of signals, we measured the signal duration, the number of oscillations, the oscillation period, and the peak frequency. As EDs had a high signal‐to‐noise ratio (SNR), a custom written semi‐automated analysis (written in Igor; Wavemetrics, Lake Oswego, OR) was used to determine the above‐described signal parameters. The sounds (50 tonal and 142 pulses) were analyzed manually using Adobe audition because they had a lower SNR and the background noise had a variety of peaks.

### 2.3 Gross morphology of the ESA

**FIGURE 2:**
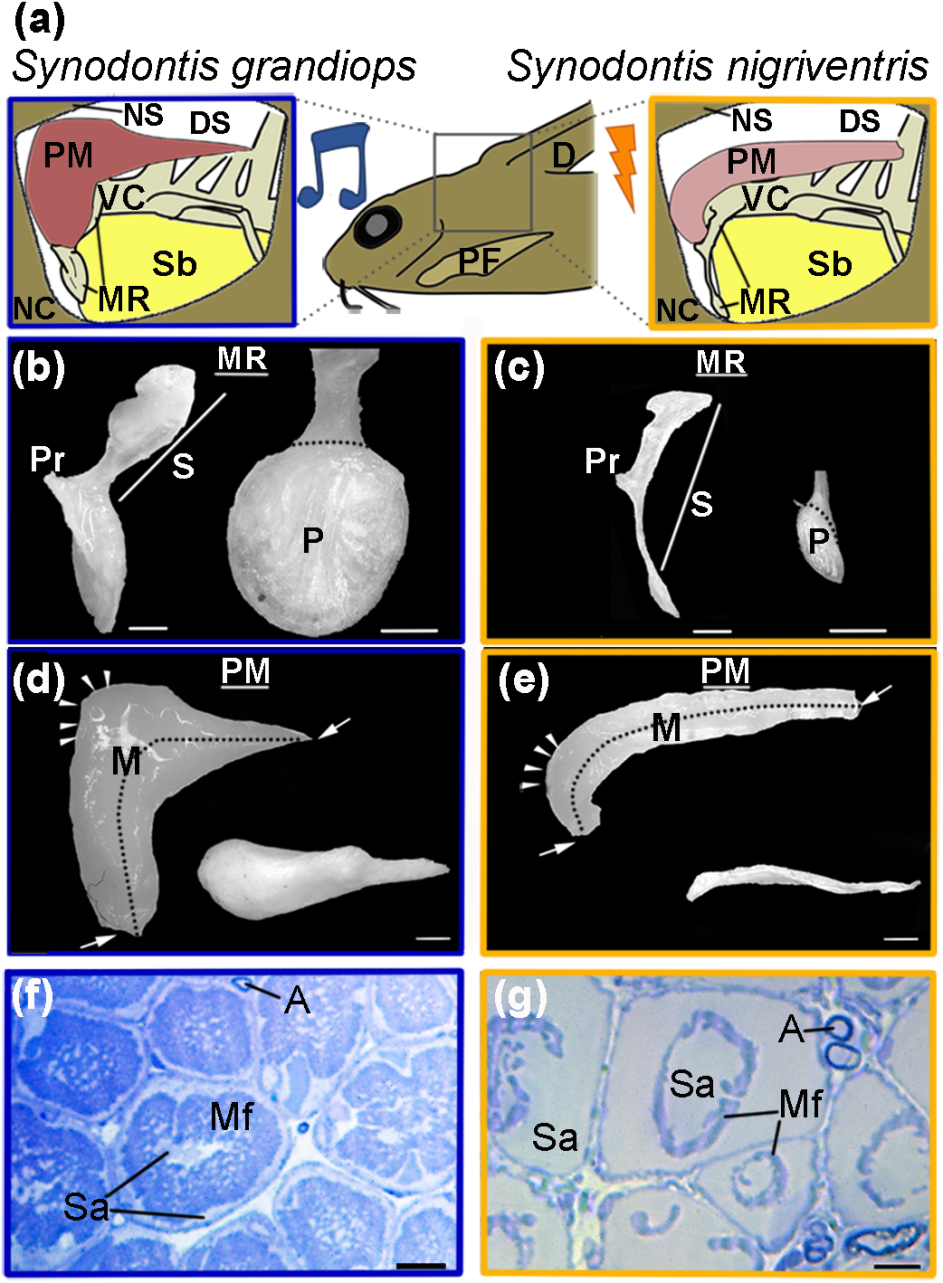
Elastic spring apparatus (ESA) in *Synodontis grandiops* and *S. nigriventris*. (a) Location (center) and schematic representation of the ESA of *S. grandiops* (left) and *S. nigriventris* (right). (b) Photographs of the Müllerian ramus of a *S. grandiops* (SL: 91 mm): Left lateral view of the Müllerian ramus (left) and medial view of the Müllerian ramus plate (right). (c) Photographs of the Müllerian ramus of a *S. nigriventris* (SL: 68 mm): Left lateral view of the Müllerian ramus (left) and medial view of the Müllerian ramus plate (P, right). (d) Photographs of the protractor muscle of a *S. grandiops* (SL: 91 mm): Left lateral view (left) and dorsal (right) views of the muscle. Arrows here and in (e) main insertion points of protractor muscle. Arrow heads here and in (e) diffuse insertion area of the protractor muscle. (e) Photographs of the protractor muscle of *S. nigriventris* (SL: 68 and 62 mm): Left lateral view (left) and dorsal (right) views of the muscle. (f) Photomicrograph of transverse section in the protractor muscle of a *S. grandiops* (scale bar: 10 μm). (g) Photomicrograph of transverse section in the protractor muscle of a *S. nigriventris* (scale bar: 10 μm). D, dorsal fin; DS, dorsal spine; M, midline (dotted line); Mf, myofibrils; P, plate of the Müllerian ramus; MR, Müllerian ramus; NC, neurocranium; NS, nuchal shield; PF, pectoral fin; PM, protractor muscle; Pr, process of the Müllerian ramus; S, stem of the Müllerian ramus; Sa, sarcoplasm; Sb, swim bladder; VC, vertebral column. White scale bars: 1 mm [Color figure can be viewed at wileyonlinelibrary.com]

Five *S. grandiops* (SL: 68 to 98 mm) and five *S. nigriventris* (SL: 52 to 72 mm), initially fixed in 7% formalin and then stored in 70% ethanol, were dissected to collect morphometric data from the protractor muscle and the Müllerian ramus (Figure 2a–e). The protractor muscle was placed in 0.1 M phosphate buffer overnight, weighed and imaged under a stereoscopic microscope (Wild M10 equipped with a MC 170 HD camera, Leica Microsystems GmbH, Wetzlar, Germany). The midline length, that is, the distance between the two main insertion points of the protractor muscle following the midline of the muscle in a lateral view, and maximal thickness of the tissue in a dorsal view were measured in ImageJ (Wayne Rasband, National Institutes of Health). The Müllerian ramus was imaged and its length, plate surface area, and stem and stem process lengths were measured following the same procedure.

### 2.4 Neuronal network identification

To visualize the neuronal network, fish were first anesthetized with 0.025% benzocaine (Sigma Aldrich Chemie GmbH, Munich, Germany) or 0.02% tricaine methanesulfonate (MS 222, Sigma Aldrich BVBA, Overijse, Belgium) dissolved in aquarium water. Long‐term anesthetic (bupivacaine, 0.25%) was applied with a soaked tissue placed on top of the surgical site and the protractor muscle was exposed. The nerve innervating the protractor muscle was cut at the level of the muscle and its proximal end‐labeled by direct application of crystals of either dextran‐rhodamine in five *S. grandiops* (SL: 67 to 88 mm) and four *S. nigriventris* (46 to 57 mm) or neurobiotin in two *S. grandiops* (54 to 58 mm) and three *S. nigriventris* (46 to 58 mm). After a survival time of two to 4 days, the fish were euthanized with an overdose of either benzocaine or MS‐222 and perfused with freshwater teleost Ringer's solution followed by a solution of 4% paraformaldehyde in 0.1 M PB. The brain was immediately dissected out of the skull, postfixed for one to 2 hr, and then stored in 0.1 M PB. Brains were subsequently embedded in 4% agar and sectioned in the transverse plane at 100 μm with a T1200S Vibratome (Leica Microsystems GmbH). Floating sections were washed in 0.5% Triton 100 (Sigma Aldrich Chemie GmbH) in 0.1 M PB (PB‐T) and incubated overnight in a 1:500 Cy3‐ (Jackson ImmunoResearch Labs, Cambridgeshire, United Kingdom, Cat# 016–160‐084, RRID:AB_2337244) or Alexa488‐streptavidin (Jackson ImmunoResearch Labs, Cat# 016‐540‐084, RRID:AB_2337249) in PB‐T solution. Sections were washed the following day three times for 30 min each time in 0.1 M PB, mounted on slides, and coverslipped using a fluorescent mounting medium (Vectashield, Vector Labs Inc., Peterborough, United Kingdom) containing 40,6‐diamidino‐2‐phenyindole (DAPI).

Every section was examined under either an epifluorescence microscope (ECLIPSE Ni, Nikon GmbH, Düsseldorf, Germany) or a confocal laser microscope (Leica Microsystems, Wetzlar, Germany) and those that contained labeled cells or fibers were imaged. Labeled somata were counted and the cell count was corrected using the Abercrombie equation (Abercrombie, 1946). The average soma diameter of every cell was measured in Adobe Photoshop CS4 (Adobe, San Jose, CA) for three specimens per species and treatment, except for two specimens of *S. grandiops* labeled with neurobiotin. Maximal projections obtained from the image stacks were cropped and optimized in Adobe Photoshop CS4 (Adobe, San Jose, CA) for illustration purposes.

### 2.5 Electromyography of protractor muscles

EMGs were recorded from three *S. grandiops* (SL: 74, 76, and 77 mm) and three *S. nigriventris* (SL: 55, 55, and 58 mm) at 25 ± 1°C (tank: 34 × 17 cm, water depth kept at ~17 cm). Bipolar recording electrodes were made using insulated nichrome (37 and 25 μm outer and inner diameters, respectively, with exposed tips of <1 mm; Clark Electromedical Instruments, Harvard Apparatus, Holliston, MA) or Teflon‐coated stainless steel (114 and 51 μm outer and inner diameters, respectively, with exposed tips of <1 mm, Science Products GmBH, Hofheimer, Germany) wires. Electrodes were inserted into each of the paired protractor muscles (reference electrode in trunk epaxial muscle) of the anesthetized (MS 222) fish. Recorded signals were amplified (high‐gain differential amplifier model 1,700; A‐M Systems, Inc., WA), digitized with an external audio interface (UltraLite mk4; MOTU, Cambridge, MA), and recorded on a laptop using Adobe Audition 2.0. Amplifying the signal 100‐fold generally provided EMGs with good SNRs and prevented clipping. Total duration, number of pulses, and pulse period were analyzed for three to five EMGs per fish and compared to the features of the associated sound or ED. For each *S. grandiops*, three EMGs associated with single‐pulse sounds were also analyzed. EMG amplitudes were not investigated because the distance between the two tips of an electrode, the extent of the electrode tips exposed, and the electrode position in the muscle were not rigorously controlled.

### 2.6 Intrinsic properties of protractor motoneurons

To investigate the intrinsic membrane properties of the motoneurons innervating the protractor muscle, fish were deeply anesthetized with benzocaine and then weighed and measured before being placed in an ice‐filled dish. The braincase was opened, and the nuchal shield and underlying muscles were removed. The brain and the rostral part of the spinal cord were removed and placed in an ice‐cold solution (in mM: 120 sucrose, 25 NaCl, 27NaHCO3, 2.5 KCl, 1.25 NaH2PO4, 3 MgCl2, 0.1 CaCl2, 25 glucose, 0.4 ascorbic acid, 3 myoinositol, and 2 Na‐pyruvate). Care was taken to remove the dura mater. The brain was then embedded in low melting agar (2% in 0.1 M PB) and cut on a vibratome (Vibrating Microtome 7,000 smz‐2; Campden Instruments Ltd., Loughborough, Leics, England) at a thickness of 200 μm in the sagittal plane while submerged in the ice‐cold solution. Slices were incubated at room temperature in a solution like that used above for dissection but with 125 mM NaCl, 1.2 mM CaCl2, and 1 mM MgCl2 and no sucrose, and oxygenated with 95% O_2_ and 5% CO_2_.

Protractor motoneuron recordings were performed under a fixed stage microscope (Axio Imager 2; Carl Zeiss Microscopy GmbH, Köln, Germany) equipped with a Dodt gradient contrast illumination and a CCD camera (Orca Flash; Hamamatsu Photonics, Hamamatsu City, Japan). Recordings were performed using an EPC 10/2 amplifier (HEKA Elektronik Lambrecht, Harvard Bioscience, Pfalz, Germany). Glass recording electrodes were filled with a solution composed of (in mM) 145 K‐gluconate, 4.5 KCl, 15 HEPES, 2 Mg‐ATP, 2 K‐ATP, 0.3 Na‐GTP, and 7.5 Na2‐phosphocreatine adjusted by adding KOH to pH 7.25. Data were acquired in current‐clamp mode at a sampling frequency of 100 kHz and low pass filtered at 3 kHz.

Protractor motoneurons from four *S. grandiops* (SL: 61 to 108 mm) and 14 *S. nigriventris* (SL: 42 to 71 mm) were patch clamped. Forty‐seven of these motoneurons in three *S. grandiops* (SL: 83 and 108 mm) and all the *S. nigriventris* were successfully filled with Alexa (488 or 546) hydrazide, imaged at high resolution under a confocal laser microscope (Leica microsystems, Wetzlar, Germany), and the image stacks used to reconstruct motoneuron somata and neurites in three dimensions using Neurolucida 360 software (MBF Bioscience, Williston, VT). The resting membrane potential was determined as well as passive membrane properties (input resistance, capacitance, time constant *Tau*), which were obtained from a − 50 pA current pulse of 0.6 s duration (1.1 s inter‐stimulation interval). Action potential (AP) firing was investigated by applying a depolarizing current pulse of varying amplitude (duration 0.6 s) until spike adaptation occurred. The rheobase (i.e., a minimal amount of current to evoke a single AP) was determined with the same stimulus (the holding current was not subtracted from the rheobase as it was similar in both species), but at current steps of 5 pA at a 1.1 s inter‐stimulus interval. Once the rheobase was found, we repeatedly stimulated the neurons 20 times at the rheobase current threshold to determine AP firing reliability and first AP latency. Action potential features such as amplitude, half‐width, afterhyperpolarization (AHP) amplitude, AHP half‐width, and afterdepolarization amplitude were obtained from individual APs that were elicited by a 0.38 ms duration pulse with a constant current intensity set by the experimenter and a 50 ms inter‐stimulation interval, as previously done in the auditory system (e.g., Ammer, Grothe, & Felmy, 2012). Stimulus duration was increased by 0.1 ms steps until an AP was fired.

### 2.7 Statistical analysis

For standardization of size, ESA morphometric data were divided by the length of the Müllerian ramus, protractor nucleus length was divided by the respective fish's SL, and neuron counts and measurements were divided by the length of the corresponding nucleus. Outliers in the data collected during the different patch‐clamp protocols were identified with the online GraphPad calculator (https://www.graphpad.com/quickcalcs/Grubbs1.cfm) and removed from the graphs and statistics. Principal component analyses obtained in the software Past 3.15 (Hammer 1999–2017) were used to explore species distribution in two‐dimensional spaces using multivariate data samples. Significance tests were combined with estimation statistics to improve our interpretation of the results. For all significance tests, the null hypothesis was that *S. grandiops* and *S. nigriventris* did not differ in a measurement. We tested this hypothesis with two‐sample Hotelling's T^2^ tests for multivariate comparisons using the “Hotelling” package of R. Subsequent comparisons of univariate data were performed with Student *t* tests (or U Mann–Whitney when the assumption of normality was not met) using Prism 5 and alpha levels rectified with the Sequential Bonferroni Correction. The effect size (mean differences and standardized Hedges' *g*) and the lower and upper bound for a 95% confidence interval were calculated online (http://www.estimationstats.com/) (see: Calin‐Jageman & Cumming, 2019; Ho, Tumkaya, Aryal, Choi, & Claridge‐Chang, 2019).

## 3 RESULTS

### 3.1 Swim bladder sounds and electric discharges

During our behavioral monitoring sessions, both species produced stridulation sounds with their pectorals (see: Parmentier et al., 2010). However, *S. grandiops* also produced swim bladder‐associated sounds, while *S. nigriventris* produced only EDs (Figure 1). *Synodontis grandiops* produced at least two types of swim bladder‐associated sounds (Table 1): pulsed (Figure 1a) and tonal (Figure 1b). Pulsed signals (*N* = 142) were brief (6 to 24 ms), broadband frequency sounds with a peak frequency at 138 ± 36 Hz (mean ± *SD*), while tonal sounds (*N* = 50) lasted between 22 and 198 ms and were multi‐harmonic with a fundamental and peak frequency of 206 ± 17 Hz. An individual emitted both types of sounds as a single event or as a part of a train of successive events with an inter‐event interval ≤ 1 s. The inter‐event intervals were highly variable, ranging from a dozen to hundreds of milliseconds. However, for some trains, single events comprised of up to 10 pulses lasted between 31 and 197 ms and had a pulse period that was relatively short and stable (22.9 ± 6.6 ms, *N* = 41). We categorized these individual events as grunt sounds.

**TABLE 1:** Comparison between the tonal sounds and electric organ discharge (ED) bursts recorded from *Synodontis grandiops* and *S. nigriventris*

	Oscillation number	Oscillation period (ms)	Fundamental frequency (Hz)
*Synodontis grandiops (S.g.)* Tonal sounds (N = 50) (mean ± *SD*)	18 ± 8	4.7 ± 0.8	206 ± 17
*Synodontis nigriventris (S.n.)* ED bursts (N = 270)	24 ± 9	9.9 ± 1.6	101 ± 16

The wave‐like EDs of *S. nigriventris* were more stereotyped and only emitted as bursts with a stable oscillation period of 9.9 ± 1.6 ms (Figure 1c; Table 1). The number of oscillations in a burst could, however, vary from 4 to 92 (*N* = 270 bursts). Like the tonal sound, the spectrogram of ED bursts showed harmonics (top row; Figure 1b, c) with a fundamental and peak frequency corresponding to the inverse of the oscillation period. Amplitude modulations within a burst could always be detected.

### 3.2 Gross morphology of the ESA

**FIGURE 3:**
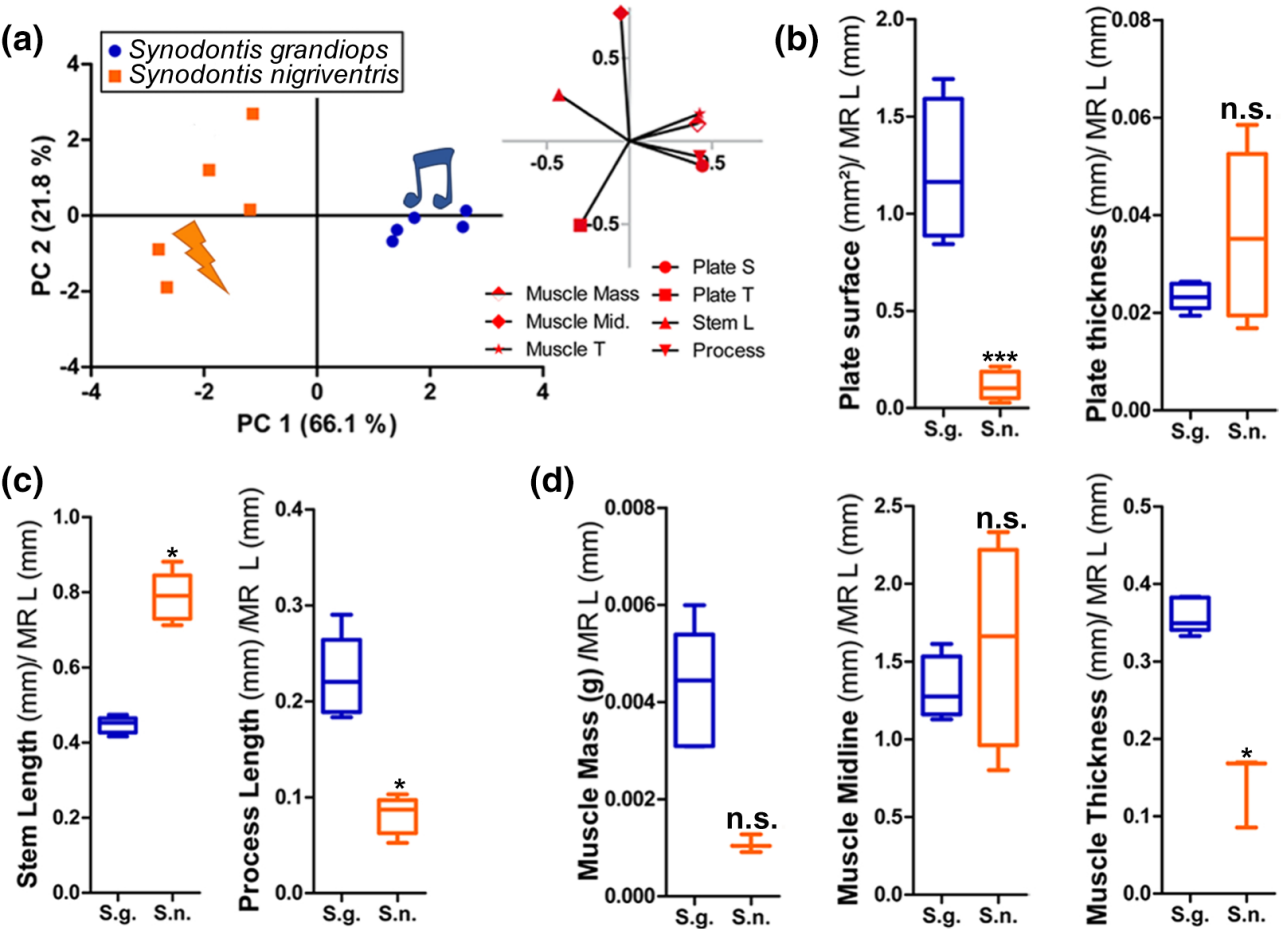
Morphometric data obtained for the elastic spring apparatus (ESA) in *Synodontis grandiops* and *S. nigriventris*. (a) Principal component analysis based on morphometric data collected from the ESA of *S. grandiops* and *S. nigriventris*. Coordinates of the individuals (left) and the variables (right) on PC1 and PC2. (b) Surface and thickness of the plate of the Müllerian ramus of *S. grandiops* and *S. nigriventris*. (c) Lengths of the stem (mm) and the process of the stem (mm) of the Müllerian ramus of *S. grandiops* and *S. nigriventris*. (d) Muscle mass (g), length of the muscle midline (mm), and maximum thickness (mm) of the protractor muscle of *S. grandiops* and *S. nigriventris*. Each variable was divided by the length of the Müllerian ramus (mm). ***: *t* test showed significant difference (*p* < .0005) between the two species. *: *t* test showed significant difference (*p* < .05) between the two species. n.s., no significant difference (*p* > .05); Muscle mid., muscle midline [Color figure can be viewed at wileyonlinelibrary.com]

Like other synodontids, the ESA in both study species was formed by the protractor muscle (PM, Figure 2a) and a modified transverse process of the fourth vertebra called the Müllerian ramus (MR, Figure 2a). The protractor muscle (PM) originated below the dorsal spine (DS) and inserted on the process (Pr) of the MR (Figure 2a–c). In its rostro‐dorsal part, the protractor muscle was also loosely attached to the neurocranium and the rostral end of the nuchal shield (NC, NS, respectively; Figure 2a). Despite these common features, there were several species differences. First, the PM was translucent in *S. nigriventris*, but whitish in *S. grandiops* (not visible in photographs of fixed muscles in Figure 2b,c). This may have resulted from the general absence of myofibrils in the PM fibers of *S. nigriventris*, which were mostly composed of sarcoplasm (SA; Figure 2f,g); also see Boyle et al. (2014). Second, the anterior and posterior half of the PM had similar sizes in *S. nigriventris*, whereas the anterior half was larger than the posterior half in *S. grandiops* (Figure 2d,e). Lastly, both species clustered in two distinct groups in a principal component analysis performed on morphometric data collected from the ESA (Figure 3a, Table S1).

Differences in the morphometric data were significant (T^2^ Hotelling = 2,471, F_7,2_ = 88.262, *p* = .0164). The MR of *S. nigriventris* had a significantly smaller plate (Figure 3b) and a longer stem, but shorter stem process (Figure 3c), while its PM was thinner (Figure 3d). The absolute values of Hedges' *g* for these variables varied between 3.83 and 6.39, which suggested extremely large interspecific differences. Using the effect size obtained from the mean differences, we estimated that the plate surface and stem processes were 91 and 64% smaller, respectively, in *S. nigriventris*, while its stem was 76% longer and its PM was 61% thinner (Table S2). Because of the Bonferroni correction, muscle mass was not tagged as significantly lighter in *S. nigriventris*. However, the effect sizes suggested a very large species effect (Hedges' *g*: −2.76); using the effect size obtained from the mean differences the PM was 75% lighter in *S. nigriventris* (Table S2). The plate thickness and the muscle midline length, on the other hand, were similar in both species (see Table S2 for the univariate tests, effect sizes and the associated bounds for 95% confidence interval).

### 3.3 EMGs of protractor muscle

**FIGURE 4:**
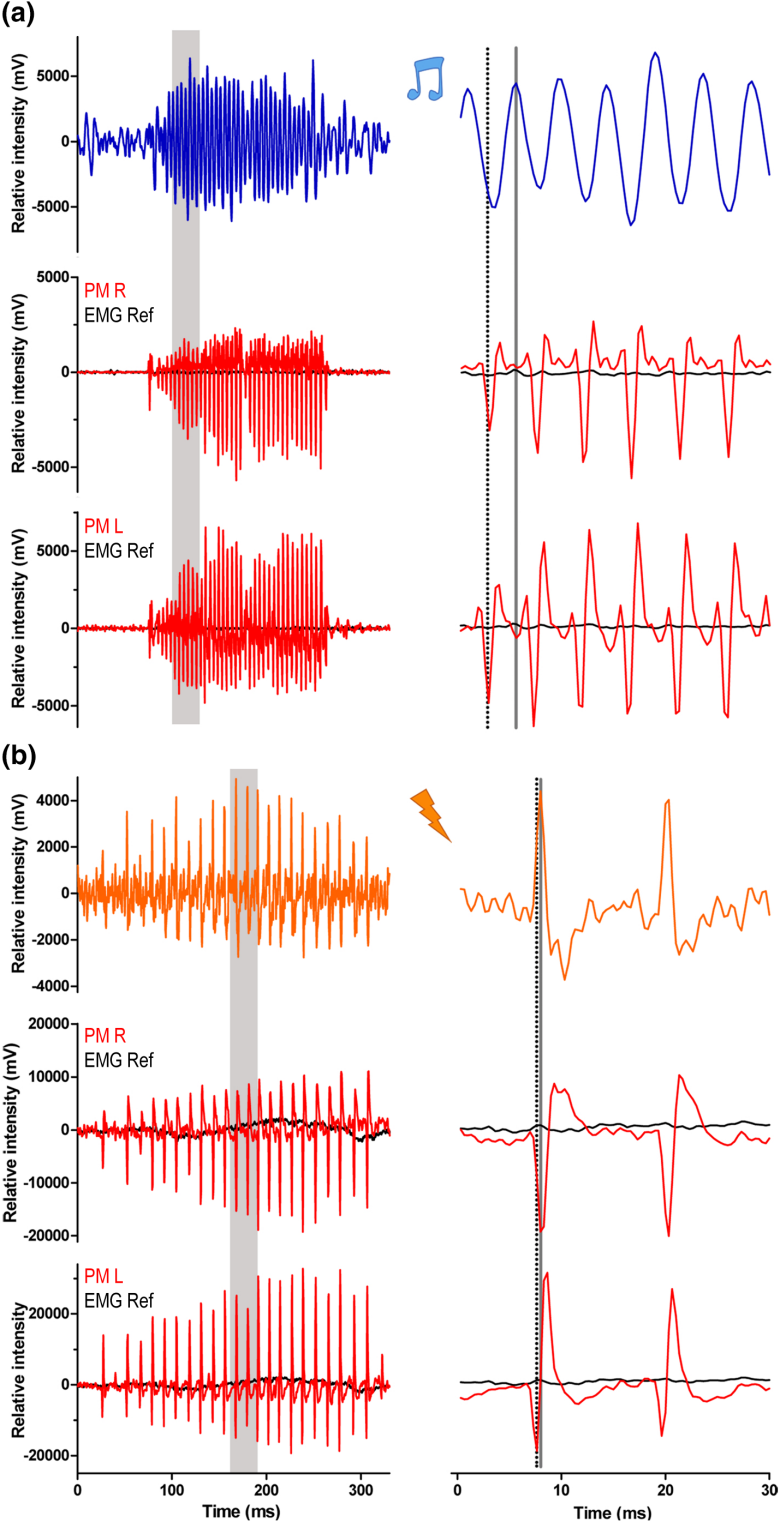
Electromyograms (EMGs) of protractor muscles in *Synodontis grandiops* and *S. nigriventris*. (a) Waveform of a tonal sound (upper trace) and trace of the associated EMGs (red) recorded from the right (middle) and left (bottom) protractor muscles. The EMGs recorded from the reference electrode are shown in black. (b) Waveform of an electric burst and trace of the associated electrical activity (red) recorded from the right (middle graph) and left (bottom graph) protractor muscles. The electrical activity recorded from the reference electrode is shown in black in the middle and bottom graphs. Right graphs show inset of the gray areas in the left graphs. The dotted black line shows the first peak in the EMG while the gray line shows the corresponding in the social signal [Color figure can be viewed at wileyonlinelibrary.com]

As shown for a tonal sound and ED (top trace; Figure 4a,b, respectively), individual, consecutive activation potentials within a bout of EMG activity were similar in amplitude for both the right (PM R) and left (PM L) protractor muscles (middle and bottom trace; Figure 4a,b; EMG ref is recording from reference electrode). Thus, the PM on both sides were activated simultaneously, ruling out the hypothesis of an alternate contraction of the left and right sonic muscle as known for sea robins (Bass & Baker, 1991; Connaughton, 2004). The number of pulses, pulse periods, and duration of the EMGs almost perfectly matched the sound of *S. grandiops* or ED of *S. nigriventris* (Figure 4, Table 2). A short delay was noticed between the activation potentials in the left and right PM and the associated sound or ED pulse. This delay was longer in *S. grandiops* than *S. nigriventris* (for the left and right protractor muscle, respectively; *S. g*.: 6.7 ± 1.2 ms and 6.8 ± 1.1 ms; *S. n*.: 1.5 ± 0.8 ms and 1.3 ± 0.9 ms). Similarly, single pulse sounds of *S. grandiops* were preceded by a single activation potential in both PMs (not shown). Here, the delays between the activation potential in left and right PM and the sound were 6.5 ± 1.9 ms and 7.3 ± 0.5 ms, respectively. The similar temporal patterns for the EMG and signal imply that both PMs are activated simultaneously and that every activation potential is responsible for a single muscle contraction in *S. grandiops* and ED in *S. nigriventris*.

**TABLE 2:** Comparison between the EMGs and signals recorded in *Synodontis grandiops* and *Synodontis nigriventris*

	*Synodontis grandiops*			
	PM L	PM R	Sound	Friedman's test
Duration (ms)	148.5 ± 65.5	144.3 ± 59.1	145.1 ± 59.1	χ^2^ = 0.67, p = 0.94
Pulse number	30.3 ± 11.5	30.0 ± 11.3	30.8 ± 11.4	χ^2^ = 1.27, p = 0.53
Pulse period (ms)	4.7 ± 0.3	4.7 ± 0.3	4.7 ± 0.3	χ^2^ = 3.71, p = 0.19
	***Synodontis nigriventris***			
	PM L	PM R	ED	Friedman's test
Duration (ms)	311.0 ± 7.9	305.1 ± 22.2	289.7 ± 19.0	χ^2^ = 4.67, p = 0.19
Pulse number	26.9 ± 3.9	26.3 ± 4.6	24.0 ± 0.6	χ^2^ = 2.67, p = 0.36
Pulse period (ms)	12.1 ± 1.5	12.0 ± 1.5	12.0 ± 1.6	χ^2^ = 3.71, p = 0.19

Abbreviations: ED, Electric discharge; PM L, EMGs recorded from the left protractor muscle; PM R, EMGs recorded from the right protractor muscle.

### 3.4 Anatomy of protractor motor and premotor neurons

#### 3.4.1 Protractor motoneurons

**FIGURE 5:**
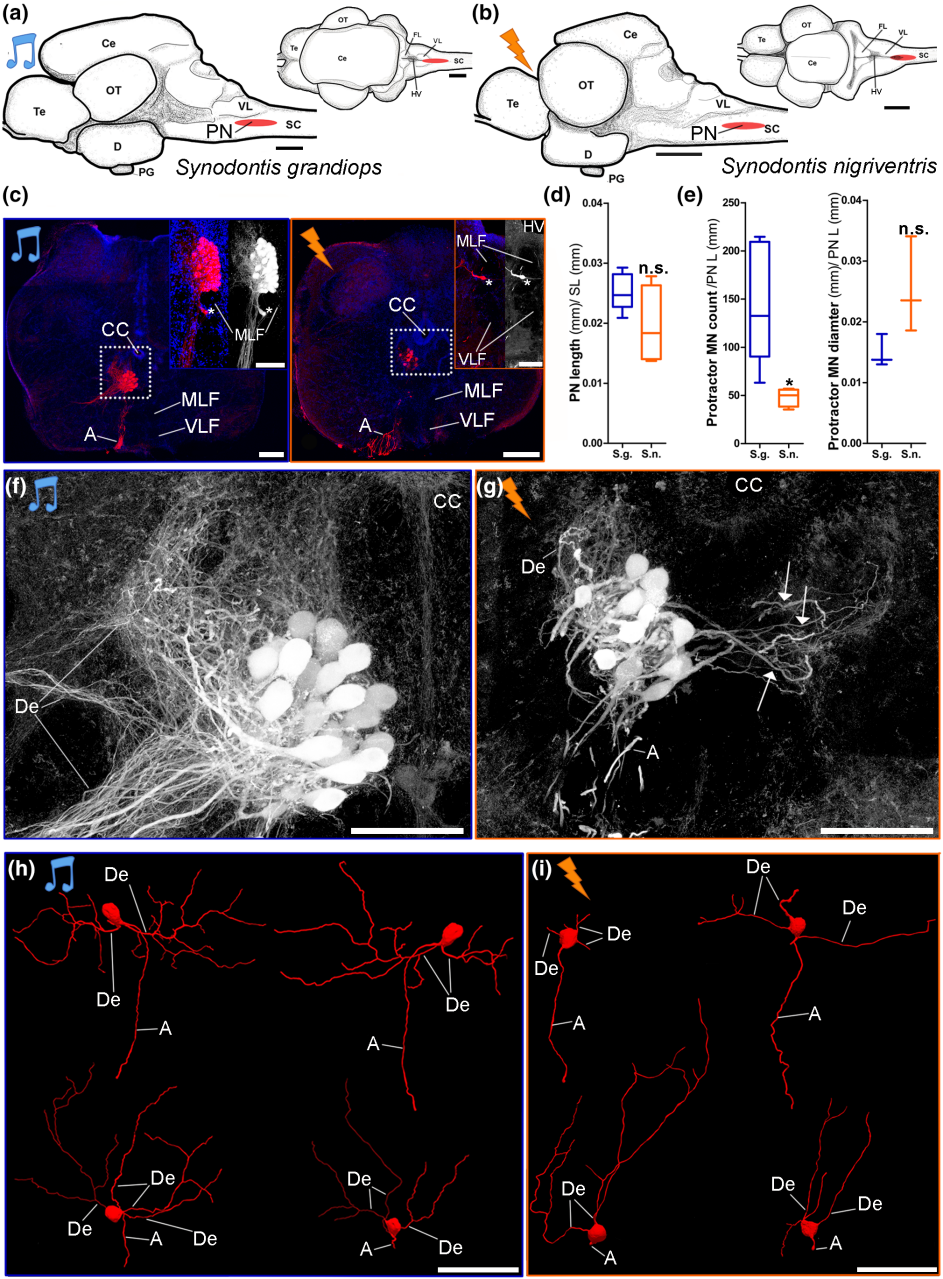
Protractor motoneurons in the brain of *Synodontis grandiops* and *S. nigriventris*. Line drawings of lateral (left) and dorsal (right) views of the brain of a (a) *S. grandiops* (SL: 67 mm) and (b) *S. nigriventris* (SL: 62 mm) (scale bars: 1 mm). The location of the protractor nucleus (PN) is shown in red. (c) Photomicrographs of motoneurons labeled with dextran‐rhodamine (red) in a transverse section of the caudal hindbrain in *S. grandiops* (left) and *S. nigriventris* (right). Nuclei are labeled with DAPI (blue). Scale bars: 200 μm. Upper right insets show protractor motoneurons (asterisks) located outside of the protractor nucleus next to the medial longitudinal fasciculus (scale bar: 100 μm). (d) Comparison of the standardized lengths of the protractor nucleus between *S. grandiops* (*N* = 7) and *S. nigriventris* (*N* = 4). (e) Comparison of the standardized counts (left) and diameter (right) of protractor motoneurons between *S. grandiops* (*N* = 7 and *N* = 3, respectively) and *S. nigriventris* (*N* = 4 and *N* = 3, respectively). (f) Higher magnification of left box shown in c. dextran‐rhodamine in greyscale. Scale bar: 100 μm. (g) Higher magnification of the right box shown in c. dextran‐rhodamine in greyscale. Scale bar: 100 μm. H, (i) partial three‐dimensional reconstructions of four motoneurons filled with Alexa (488 or 546) hydrazide during the patch‐clamp recordings of *S. grandiops and S. nigriventris*, respectively. These motoneurons were imaged at high resolution under a confocal laser microscope and reconstructed using the software Neurolucida 360. Scale bar: 100 μm. Every variable was size standardized. *: Significant difference (*p* < .05). n.s.: not significant (*p* > .05). Arrows: dendrites crossing the midline. A, axons; CC, central canal; Ce, cerebellum; D, diencephalon; De, dendrites; FL, facial lobe; HV, fourth ventricle; MLF, medial longitudinal fasciculus; OT, optic tectum; PNL, protractor nucleus length; PG, pituitary gland; Te, telencephalon; SC, spinal cord; VL, vagal lobe; VLF, ventral longitudinal fasciculus [Color figure can be viewed at wileyonlinelibrary.com]

Dextran rhodamine, which does not pass through gap junctions and thus only labels motoneurons (e.g., Bass et al., 1994; Song, Ampatzis, Björnfors, & El Manira, 2016; Viana, Gibbs, & Berger, 1990), labeling of the protractor muscle on one side of the body allowed the identification of the protractor motoneurons and the delineation of the protractor nucleus (PN) (Figure 5a,b). The PN is not referred to as only a motor nucleus because it also includes one small population of premotor neurons (PN1, see below).

The PN had a dense cluster of motoneurons located near the midline and above the medial longitudinal fasciculus (MLF, Figure 5c). An average of 224 ± 55 and 41 ± 14 motoneurons were labeled in the PN of *S. grandiops* (*N* = 5) and *S. nigriventris* (*N* = 4), respectively. Only ipsilaterally labeled neurons were detected (Figure 5c) with few (4 ± 5 neurons in *S. grandiops* and 1 ± 1 neuron in *S. nigriventris*) located adjacent to the PN, lateral to the MLF (asterisks in Figure 5c). PN length was 1960 ± 357 μm and 976 ± 252 μm in *S. grandiops* (*S.g*.) and *S. nigriventris* (*S.n*.) (Figure 5d). Normalization of the data using body size showed that *S. grandiops* had significantly more protractor motoneurons than *S. nigriventris* (Figure 5e left, Table S3). The effect size for this variable was large (Hedges' *g*: −1.72) and suggested that *S. nigriventris* had 65% fewer motoneurons (Table S3). Motoneuron somata had an average diameter of 26 ± 4 μm and 22 ± 4 μm in *S. grandiops* and *S. nigriventris*, respectively (Figure 5e right). The differences in PN length and motoneuron diameter were not significant (Figure 5d,e; Table S3). Even though the effect sizes suggested that the PN was smaller and the motoneurons were larger in *S. nigriventris*, the results provided by the significance tests were supported by the fact that the ranges of the 95% confidence intervals were much larger than the effect sizes.

Dextran‐biotin labeling showed for both species that protractor motoneuron axons (A) projected ipsilateral and ventral, and dendrites (De) projected dorsal and lateral into the contralateral PN (Figure 5c,f,g). Three‐dimensional reconstructions of individual motoneurons filled during the patch‐clamp experiments further showed a single nonbranching axon emerging from the soma and up to four primary dendrites (A and De, respectively; Figure 5h,i). A qualitative comparison suggested that the primary dendrites of *S. grandiops* were more branched. Dorsal dendritic fields were robust in both species. Contralateral and lateral dendritic fields were also observed in both species, but lateral ones were more prominent in the rostral PN in *S. grandiops* (Figure 5f) and contralateral ones in *S. nigriventris* (Figure 5g).

#### 3.4.2 Premotor neurons

**FIGURE 6:**
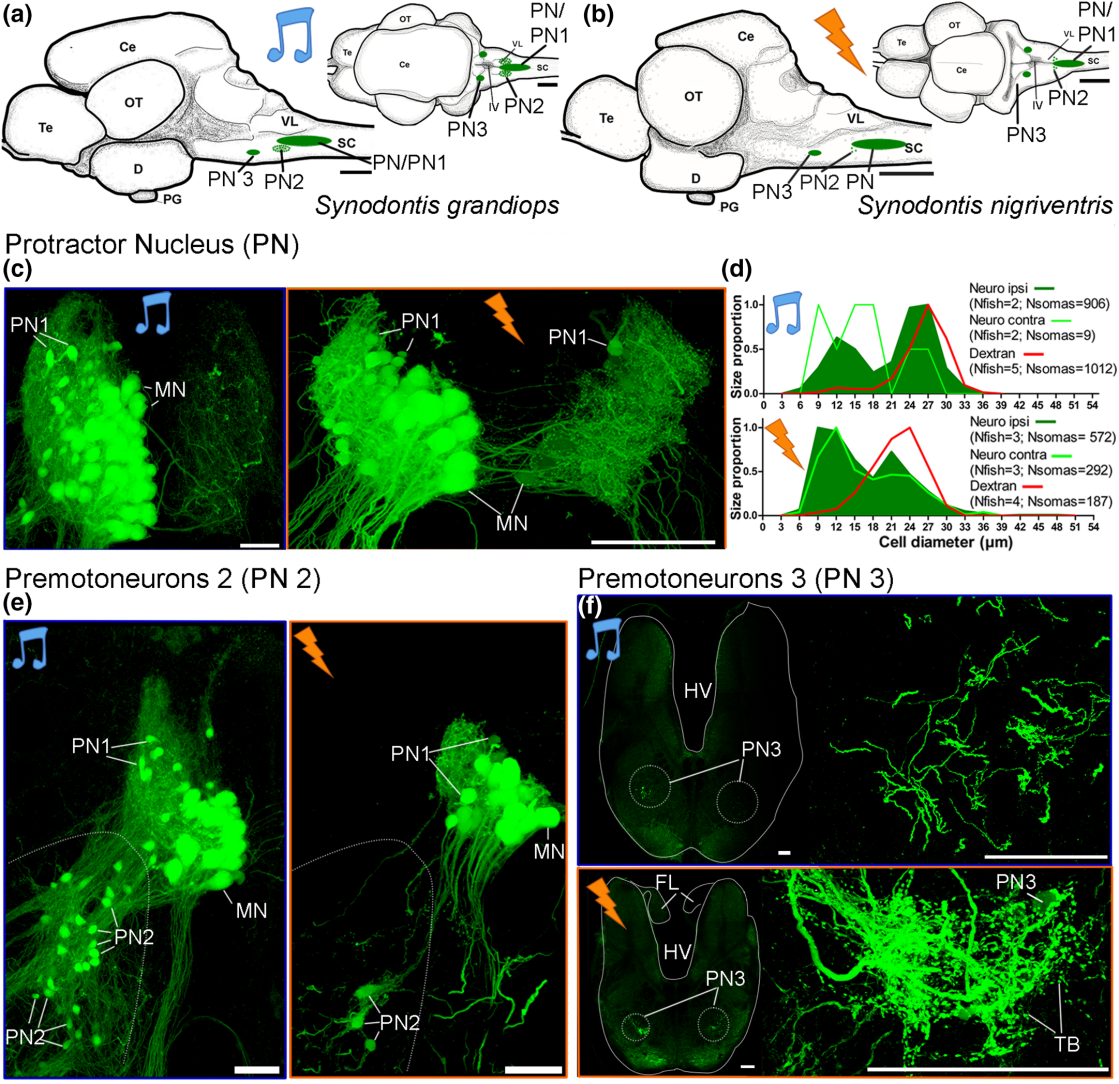
Protractor motor and premotor nuclei in *Synodontis grandiops* and *S. nigriventris*. Line drawings of a brain in lateral (left) and dorsal (right) views showing location of the neuron populations of a *S. grandiops* (a) and *S. nigriventris* (b) after the application of neurobiotin on peripheral nerve branches labeled at the level of the protractor muscle. (c) Neurobiotin‐labeled motoneurons (MN) and type I premotor neurons (PN1) in the ipsilateral protractor nucleus (PN) of a *S. grandiops* (left) and a *S. nigriventris* (right). (d) Histogram showing the distribution (normalized to 1) of neuron sizes (diameter in μm) in the PN after the application of dextran rhodamine (red) or neurobiotin (ipsilateral: Dark green; contralateral: Light green) in *S. grandiops* (top) and *S. nigriventris* (bottom). (e) Type 2 premotor neurons in *S. grandiops* (left) and *S. nigriventris* (right). (f) Left: low power photomicrographs of hindbrain showing location of type 3 premotor neurons that are rostral to PN in *S. grandiops* (top) and *S. nigriventris* (bottom). Right, Higher magnification views of PN3. Other abbreviations: Ce, cerebellum; D, diencephalon; FL, facial lobe; HV, fourth ventricle; OT, optic tectum; PT, pituitary gland; Te, telencephalon; SC, spinal cord; TB, putative terminal boutons; V, ventral fasciculus; VL, vagal lobe [Color figure can be viewed at wileyonlinelibrary.com]

Three populations of premotor neurons were identified in both species (PN1‐3, Figure 6a,b) following the labeling of one protractor muscle with neurobiotin, a tracer that is sufficiently small to pass through gap junctions (Bass et al., 1994). As previously described (Ladich & Bass, 1996), Type 1 neurons (PN1) were located in the PN (Figure 6c). These neurons were smaller in size compared to protractor motoneurons identified with dextran‐rhodamine (Figure 5) and most were in the dorsolateral PN (Figure 6c). Consequently, the measurement of PN cell sizes in the neurobiotin experiments revealed a double‐peaked histogram (Figure 6d). While the first peak predominantly resulted from PN 1, the second likely originated from motoneurons as it overlapped measurements obtained from the labeling of motoneurons with dextran‐rhodamine (Figure 6d). In both species, the diameter of PN1 neurons was approximately half that of motoneurons (Figure 6d).

PN2 neurons were located ventrolateral to the rostral PN (also see Ladich & Bass, 1996) in both species and appeared to be far more numerous in *S. grandiops* (Figure 6e). Fibers projecting rostrally from the PN2 could be followed to a few lightly labeled PN3 neurons forming a small bilateral nucleus in the ventrolateral reticular formation (Figure 6a,b,f). Only a few neurons were labeled in PN3, but a dense mesh of fibers and putative terminal boutons were observed. Contralaterally labeled fibers must have originated from PN1 and/or PN2 (Figure 6c).

While the general pattern of the premotor organization was shared between the two species, neurobiotin tracings showed some differences. In *S. nigriventris*, many protractor motoneuron and PN1 somata (88.7 ± 28.5 cells) were also labeled in the contralateral PN (Figure 6d), though more lightly labeled than on the ipsilateral side. Contralateral labeling appeared extremely rare (3.9 ± 1.7 cells) in *S. grandiops*. This most likely explained why the size distribution of the somata showed three peaks for the contralateral labeling in *S. grandiops*, while only two peaks were observed for the other neurobiotin labeling (Figure 6d). Finally, we found transneuronal labeling of some PN3 neurons in *S. nigriventris*, but not in *S. grandiops*. The size of PN3 neurons (12.1 ± 2.6 μm; *N* = 26) was similar to that of PN1 and PN2. Despite these small differences, our experiments showed that the protractor muscle of these two species is controlled by the same motor‐premotor circuit.

### 3.5 Intrinsic properties of protractor motoneurons

Having established the basic anatomical pattern, we next asked whether neurophysiological differences could be observed at the level of individual neurons. Patch‐clamp recordings from 14 motoneurons in three *S. grandiops* (*S. g*.) and 12 in four *S. nigriventris* (*S. n*.) were highly stable with low holding current (mean ± *SD*: −5 ± 56 pA). Motoneurons in both species showed no spontaneous activity and APs could only be evoked with considerable amounts of current (hundreds of pA) shortly after current injection onset.

**FIGURE 7:**
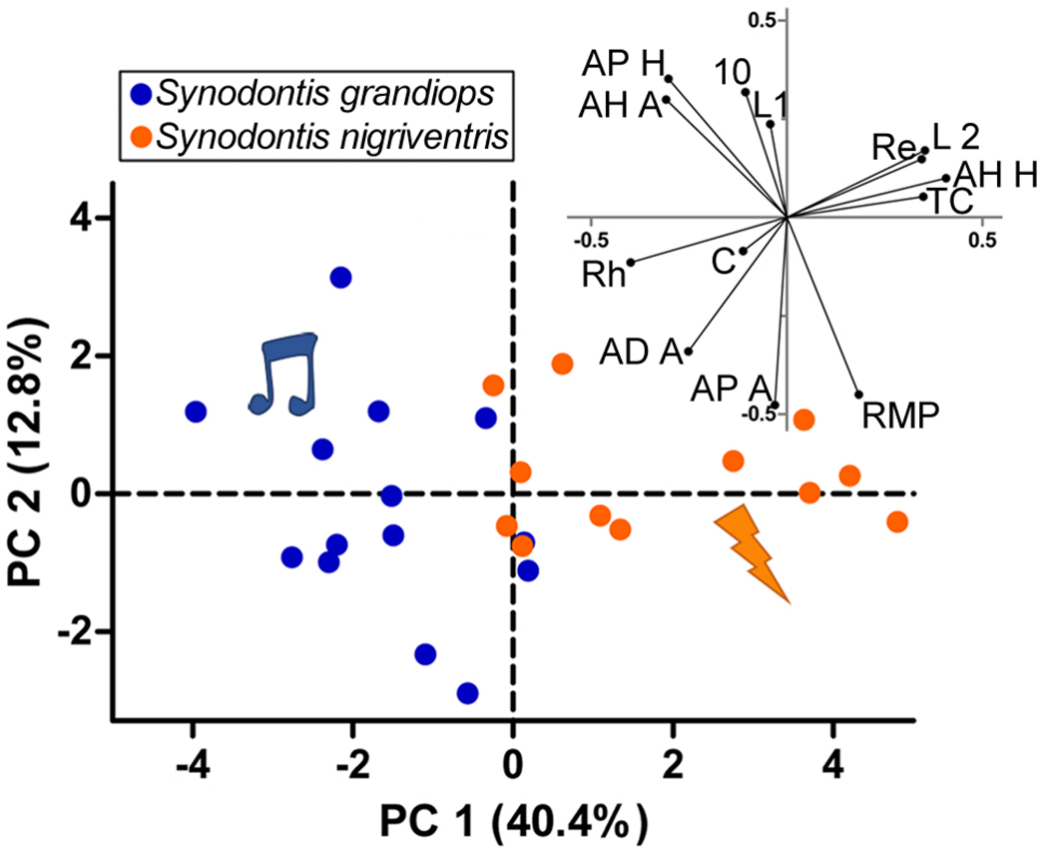
Principal component analysis based on the electrophysiological data obtained from the protractor motoneurons of *Synodontis grandiops* and *S. nigriventris*. Coordinates of the individuals (left) and loadings of the variables (right) on PC1 versus PC2. Blue: *S. grandiops*. Orange: *S. nigriventris*. 10: Number of action potentials for a stimulus 10% over the rheobase. AP A, action potential amplitude; AP H, action potential half‐width. AD A, Afterdepolarization amplitude; AH A: afterhyperpolarization amplitude; AH H, Afterhyperpolarization half‐width; C, capacitance. L1: latency measured with long pulses at the rheobase; L2, latency measured on a short pulse; Re, resistance; Rh, rheobase; RMP, resting membrane potential; TC, time constant *Tau* [Color figure can be viewed at wileyonlinelibrary.com]

**FIGURE 8:**
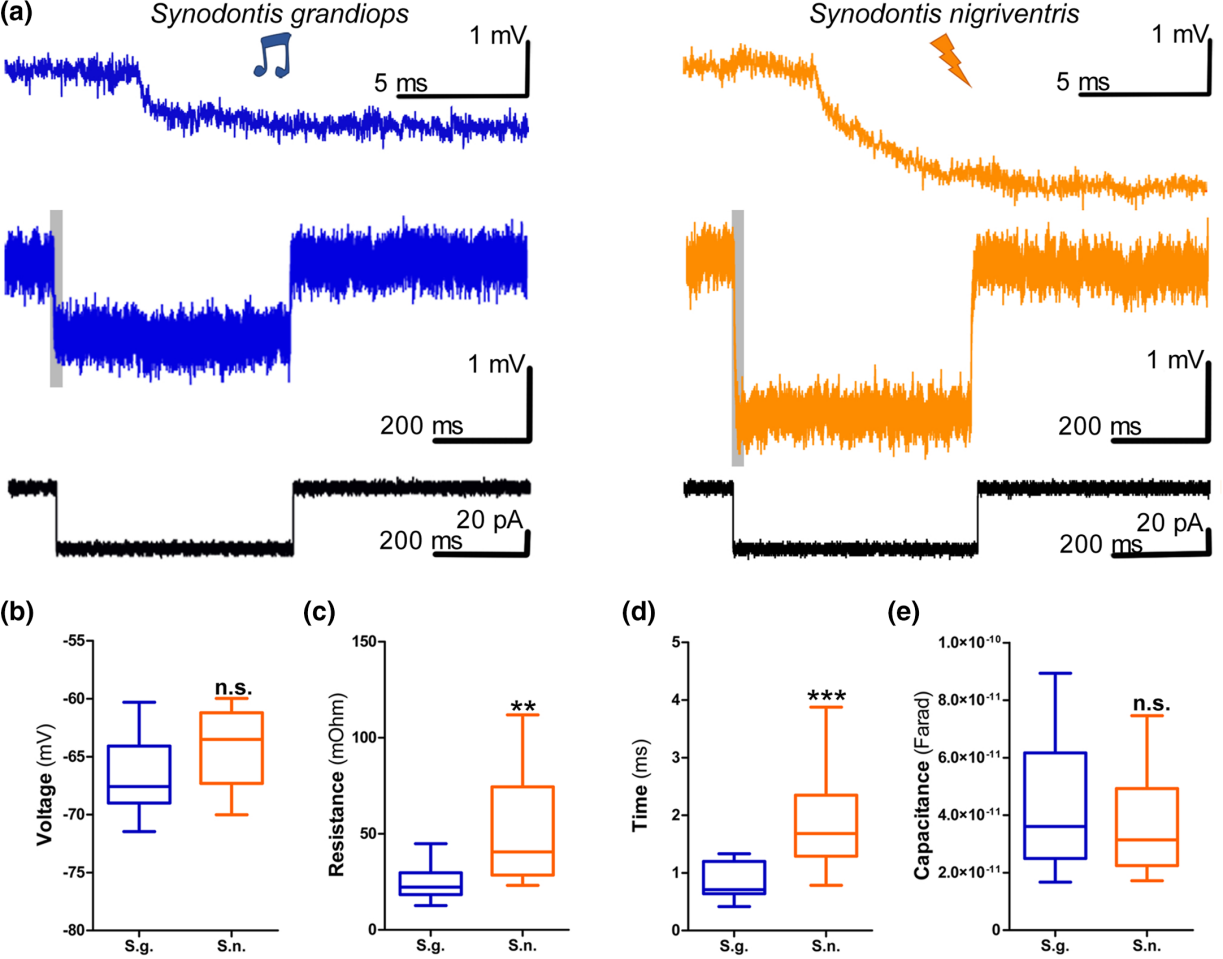
Passive properties of the protractor motoneurons of *Synodontis grandiops* and *S. nigriventris*. (a) Responses of a *S. grandiops* (blue traces, left) and a *S. nigriventris* (orange traces, right) motoneuron to a hyperpolarizing pulse (black traces, bottom). Top traces are higher magnifications of the gray areas in the middle traces. (b–e) Quantitative measures from *S. grandiops* (blue) and *S. nigriventris* (orange) motoneurons for resting membrane potential (b), resistance (c), time constant *Tau* (d), and capacitance (e) obtained from responses to long hyperpolarizing pulses [Color figure can be viewed at wileyonlinelibrary.com]

A multivariate comparison of 13 neurophysiological variables (Figure 7; Tables S4, S5) showed significant species differences in some membrane properties (T^2^ Hotelling = 201.36, F_13,12_ = 7.75, *p* = .0005). Four variables measured passive membrane properties (Figure 8a). The resting membrane potential varied from −71 to −60 mV, but did not differ (Figure 8b; Table S4) between *S. grandiops* and *S. nigriventris* (−66.5 ± 3.4 mV and − 64.3 ± 3.5 mV, respectively), and the standardized effect size was small (Hedges' *g*: 0.6). The input resistance of the individual motoneurons (24.4 ± 8.8 mΩ and 52.2 ± 27.8 mΩ, respectively) and the time constant *Tau* (0.9 ± 0.3 ms and 1.9 ± 0.9 ms, respectively) were significantly larger in *S. nigriventris* (Figure 8c,d). Effect sizes for the input resistance and the time constant were large (Hedges' *g*: 1.38 and 1.59, respectively) and the “Mean Difference” method estimated that they increased by 114 and 120%, respectively. Those differences were, however, not reflected in motoneuron capacitance (Figure 8e, Table S4).

**FIGURE 9:**
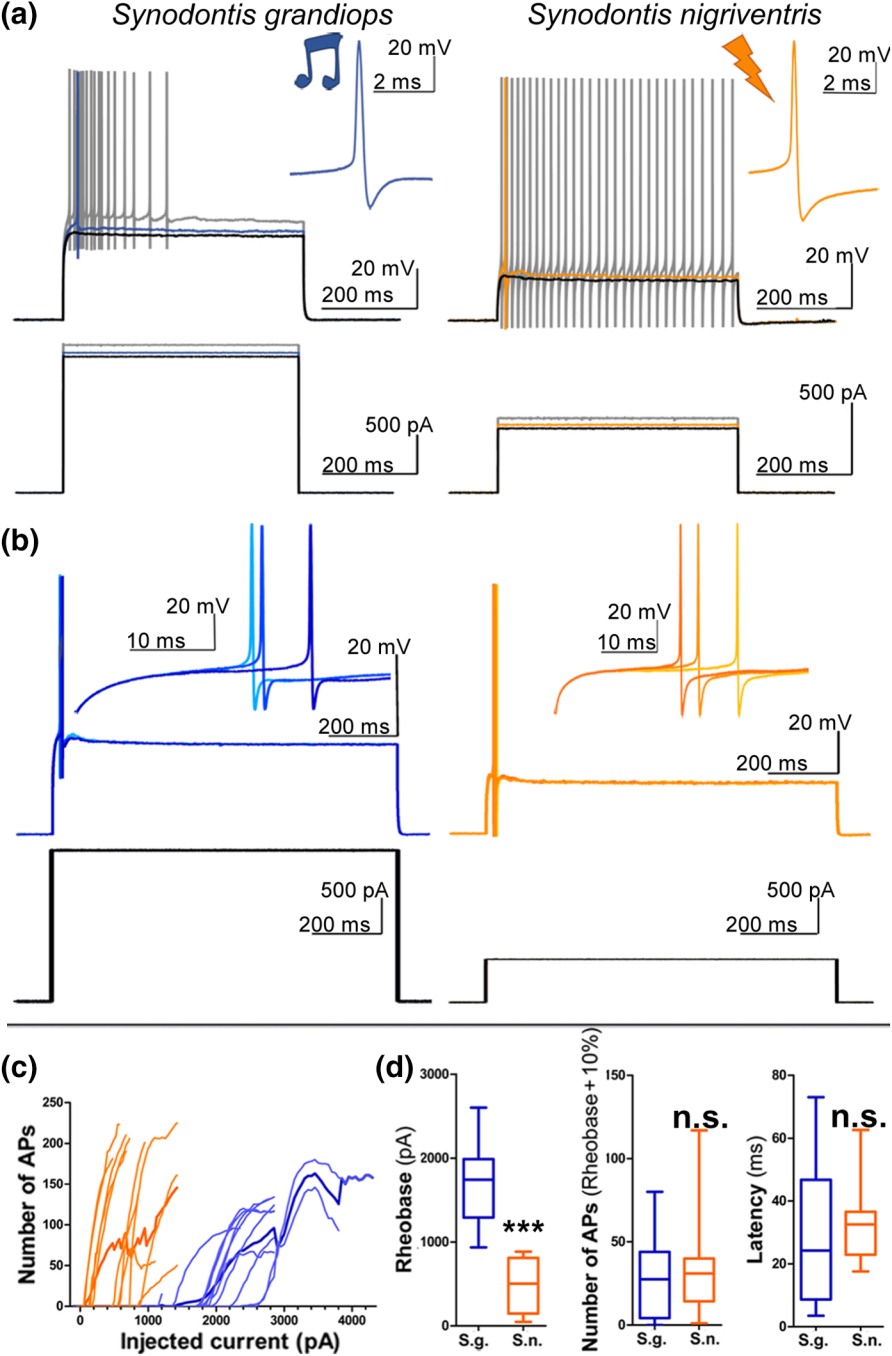
Rheobase and stimulus to action potential latency of the protractor motoneurons of *Synodontis grandiops* and *S. nigriventris*. (a) Intracellular traces from *S. grandiops* (left) and *S. nigriventris* (right) of a protractor motoneuron (top) and stimuli (bottom) at the rheobase (blue/orange traces), 25 pA under the rheobase (black), and 50 pA over the rheobase (gray) using long stimuli. (b) Three intracellular traces from *S. grandiops* (left) and *S. nigriventris* (right) of a protractor motoneuron (top) and the associated stimuli (bottom) at the rheobase. (c) Number of action potentials (APs) recorded in *S. grandiops* (blue) and *S. nigriventris* (orange) depended on the injected current using the protocol shown in (a). (d) Box plots in *S. grandiops* (blue) and *S. nigriventris* (orange) of the rheobases, number of APs at stimuli 10% over the rheobase, and latency [Color figure can be viewed at wileyonlinelibrary.com]

The remaining nine variables measured active properties. Using suprathreshold stimuli, both species tended to fire trains of APs (Figure 9a). The number of APs in trains did not differ for stimuli 10% over the respective rheobases and the effect size was negligible (Figure 9c, Table S4). While the rheobase was relatively high in both species, it was significantly lower in *S. nigriventris* compared to *S. grandiops* (460 ± 328 pA and 1,705 ± 468 pA, respectively) (Figure 9d, Table S4). The standardized effect size was very large (Hedges' *g*: −2.94) and the rheobase was estimated to be 68% lower in *S. nigriventris* (Table S4). Motoneuron firing precision was tested by measuring the AP latency for repeated stimulus applications at the rheobase threshold current (Figure 9b). Both species showed relatively short (28.9 ± 23.4 ms in *S. grandiops* and 32.5 ± 12.4 ms in *S. nigriventris*) latencies (Figure 9d) that did not differ statistically. Again, the effect size was negligible (Table S4).

**FIGURE 10:**
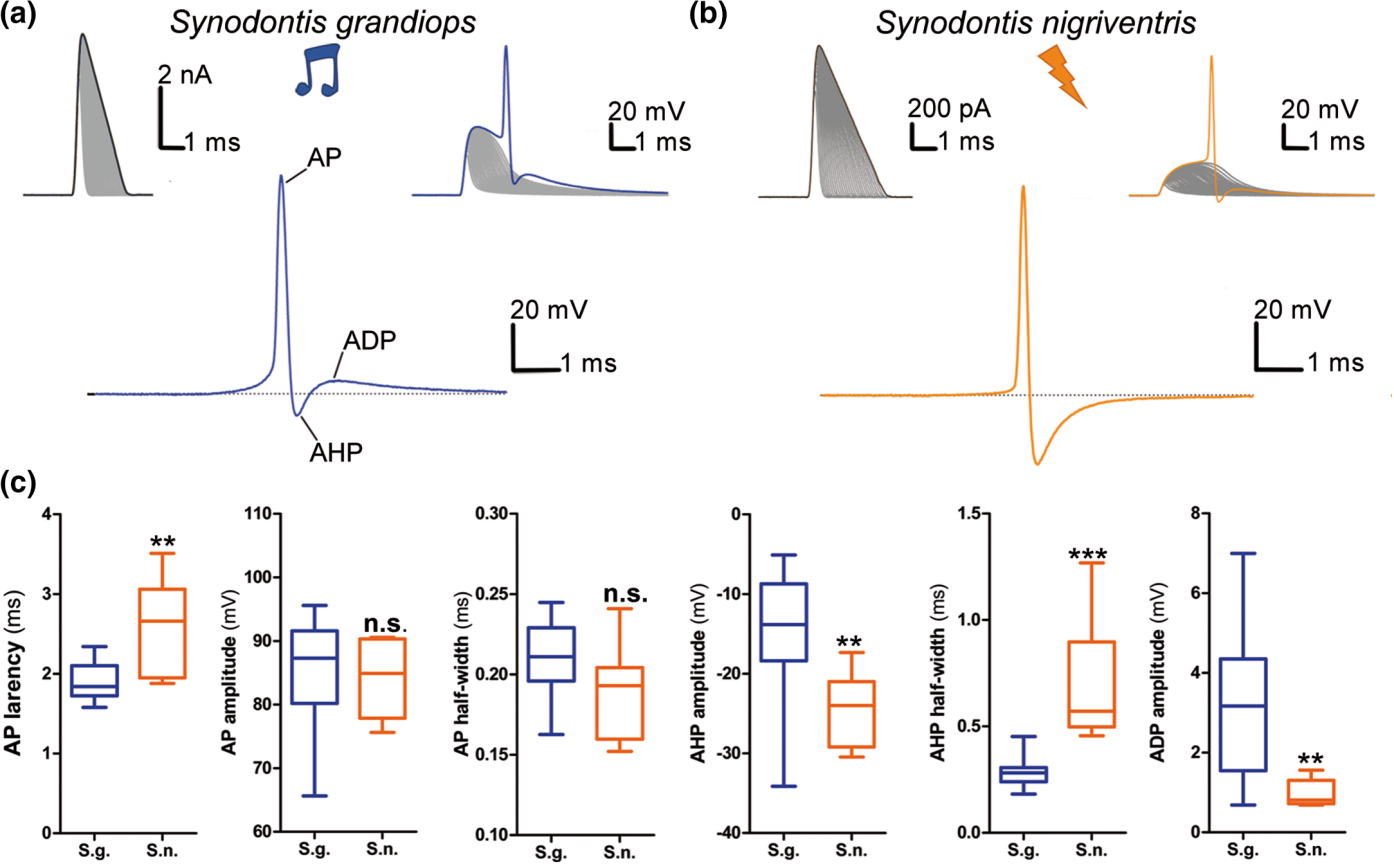
Intrinsic properties of protractor motoneuron action potentials in *Synodontis grandiops* and *S. nigriventris*. First action potential (AP) recorded in *S. grandiops* (a) and *S. nigriventris* (b) during the short stimulus protocol: Stimuli (top left), recordings (top right), and the trace obtained by subtracting the stimulus to the first recording with an AP (bottom). (c) Box plots for *S. grandiops* (blue) and *S. nigriventris* (orange) of the AP amplitude, AP half‐width, AP latency, afterhyperpolarization (AHP) amplitude, AHP half‐width, and afterdepolarization (ADP) amplitude [Color figure can be viewed at wileyonlinelibrary.com]

Action potential latency to a current injection mimicking a strong synaptic input was also measured (Figure 10a,b) and, although it was very short in both species (1.9 ± 0.24 ms in *S. grandiops* and 2.57 ± 0.6 ms *S. nigriventris*), it differed significantly at their respective thresholds (Figure 10c, Table S4). Interestingly, the AP was fired in the downward slope of the membrane potential following current injection in *S. grandiops* (see Figure 10a), which might be due to either the membrane properties or a longer distance of the axon hillock from the soma in *S. grandiops* compared with *S. nigriventris*. *S. grandiops* and *S. nigriventris* had similar AP amplitudes (84.4 ± 9.6 mV and 84 ± 6 mV, respectively) and half‐widths (0.21 ± 0.03 ms and 0.19 ± 0.03 ms, respectively) (Figure 10c, Table S4). The AHP was significantly larger (−24.3 ± 4.3 mV and − 14.8 ± 8.7 mV, respectively) and longer (0.72 ± 0.28 ms and 0.29 ± 0.07 ms, respectively) in *S. nigriventris* compared with *S. grandiops* (Figure 10c, Table S4). The amplitude of the afterdepolarization (ADP), on the other hand, was larger (1 ± 0.3 mV and 3.3 ± 1.9 mV, respectively) in *S. grandiops* (Figure 10c, Table S4). Action potential features that showed interspecific differences also had a large (|Hedges' *g*| > 1.32) standardized effect size (Table S4). Among them, the largest Hedges' *g* (2.08) was obtained for the half‐width of the AHP which was estimated to be 145% larger in *S. nigriventris* (Table S4).

## 4 DISCUSSION

Comparative studies suggest that sound production is the ancestral character state for the protractor motor system of synodontid catfish (see Boyle et al., 2014). The results presented here would then suggest that both anatomical and neurophysiological modifications in the protractor muscle and its associated hindbrain premotor‐motor network underlie the evolution of a novel, weakly electric signaling channel among synodontids. The goal of this study was to identify characters linked to being either sonic or electrogenic, or common to both behaviors. We recognize, however, that limiting our approach to a single sonic‐only and a single electrogenic‐only synodontid species cannot account for the full range of interspecific variance. Consequently, the presence and functional significance of the characters that we identified here need to be investigated in other synodontid species to more completely assess their contribution to sonic and/or electrogenic signal production.

### 4.1 Shared characters between vocal and electrogenic species

Shared characters between the neural control of sonic and electric signaling among synodontids likely relate to similar functional demands (Bass, 1989; Bass & Baker, 1997; Bass & Zakon, 2005). First, electric and vocal signals often require synchronous firing of neurons (Bass, 2014; Bass et al., 1994; Bennett, 1971; Carlson, 2006). Synchronization of neuronal activity has been, in part, attributed to gap junction coupling of neurons, a feature found in electric (Bennett, 1971; Bennett, Nakajima, & Pappas, 1967; Bennett, Pappas, Aljure, & Nakajima, 1967; Bennett, Pappas, Giménez, & Nakajima, 1967; Carlson, 2006; Elekes & Szabo, 1985) and sound‐producing (Chagnaud, Zee, & Baker, 2012; Pappas & Bennett, 1966) fish. Our and previous (Ladich & Bass, 1996) transneuronal tracing experiments are highly suggestive of gap junctional coupling in the protractor circuit in both *S. grandiops* and *S. nigriventris*. Although the presence of gap junctions in these synodontids seems likely given the extensive labeling that we and Ladich and Bass (1996) observed between motor and premotor populations, specific uptake mechanisms for biotin‐derived compounds at chemical synapses should not be discounted (see discussion in Bass et al., 1994). Conversely, an inherent limitation of this tracing method is that additional premotoneurons connected only with chemical synapses to the motoneurons may not be visualized if there is no such mechanism.

Second, the neural networks for vocal and electric signal production often generate a temporally precise activation pattern (Bass, 2014; Bass & Baker, 1997; Bass & Zakon, 2005). In the frog *Xenopus laevis* (Yamaguchi, Kaczmarek, & Kelley, 2003), for instance, vocal motoneurons fire only under large depolarization currents and with short onset latencies, features that are well suited to follow rhythmic activity. Vocal motoneurons in midshipman fish (Chagnaud et al., 2012) and both of the synodontid species investigated here did not show spontaneous activity, a characteristic onset firing property, and a low excitability, all features in line with neurons adapted to precise firing.

Lastly, in some species, the peripheral organs generating vocal and electric communication signals follow the fast synchronous oscillations of the neural network (Chagnaud et al., 2012; Rome, Syme, Hollingworth, Lindstedt, & Baylor, 1996; Unguez & Zakon, 1998). Calcium uptake by the sarcoplasmic reticulum is a major limiting factor when it comes to contraction speed in vertebrates (Rome & Lindstedt, 1998). Compared with locomotor muscles, sonic muscles have a similar rate of calcium uptake, but a higher content of sarcoplasmic reticulum allowing them to overcome this limitation (Bass & Marchaterre, 1989; Fawcett & Revel, 1961; Feher, Waybright, & Fine, 1998; Kéver, Boyle, Dragičević, Dulčić, & Parmentier, 2014; Millot & Parmentier, 2014; Rome & Lindstedt, 1998). Electrocytes generally have fewer myofilaments (Bass et al., 1986; Schwartz, Pappas, & Bennett, 1975), likely because they do not need to generate any movement to generate their electric fields. Fibers in the protractor muscle of *S. grandiops* and *S. nigriventris* have the same general morphology of sonic fibers and electrocytes, respectively (Boyle et al., 2014), suggesting convergent evolution. We thus propose that some synodontid species have evolved a novel communication channel, namely weakly electric communication, using the highly conserved ESA and its associated neural circuitry because both electric and acoustic signals involve fast, synchronous activation of the peripheral target generating the signal.

### 4.2 How was the transition to electric signaling achieved in synodontids?

Peripheral differences are easily related to the mode of communication. The sound producer in this study, *S*. *grandiops*, had a larger muscle with higher proportions of myofibrils and a larger bony plate at the level of the Müllerian ramus, all of which is well adapted for vibrating the swim bladder. For the ED producer, *S. nigriventris*, the lower proportion of myofibrils can be related to a lack of driving force acting on a sonic system. Thus, the muscle and plate of the Müllerian ramus are smaller. Electrocytes generally have a higher degree of surface proliferation and a polarized innervation and distribution of ion channels (Bass et al., 1986; Caputi, Carlson, & Macadar, 2005; Schwartz et al., 1975). Similar adaptions of the protractor muscle cells should be expected in *S. nigriventris*, but have not yet been investigated.

Despite a similar organization of the neural network, the ED producer (*S. nigriventris*) showed fewer motoneurons. This character might further enhance neuronal synchronization, as it has been suggested that decreasing the number of neurons in the pacemaker nucleus could lower the ED's coefficient of variation in gymnotiform fishes (Crampton, 2006). This notion is strengthened by strongly electric catfish, in which only a single pair of electromotoneurons innervate the entire electric organ comprised of millions of electrocytes (Bennett, Nakajima, & Pappas, 1967; Janetzko, Zimmermann, & Volknandt, 1987). The lower number of protractor motoneurons in the electric species studied here could also be related to the smaller size of its protractor muscle, which could further relate to the number of muscle fibers/electrocytes. Comparisons with other synodontids (or even mochokid) species producing either swim bladder sounds or EDs would help resolve this matter.

*Synodontis grandiops*, the sonic species studied here, also had more neurons in PN2 than *S. nigriventris*. PN3, on the other hand, was not clearly labeled in *S. grandiops*. Since there are currently no electrophysiological recordings from PN1‐3, these differences are difficult to interpret in a functional context. It is also difficult to explain the stronger labeling in *S. nigriventris* of contralateral motoneurons, premotor neurons, and PN3. The most parsimonious explanation at this point would be that it further improves bilateral, synchronous firing of motoneurons and premotor neurons in *S. nigriventris*, but there is currently no evidence supporting the hypothesis that electric signaling requires a higher level of bilateral synchronization than the production of swim bladder sounds for any fish species.

Based on our EMG experiments, the higher fundamental frequency, or pulse repetition rate, of the *S. grandiops* tonal sounds compared with the *S. nigriventris* EDs is associated with a higher rate of simultaneous‐occurring activation potentials in the paired protractor muscles (vs. alternate muscle contraction). In *S. grandiops*, the action potential latency and AHP were shorter, but the AHP was larger suggesting that the *S. grandiops* protractor sonic motoneurons are tuned to fire successive action potentials faster than the *S. nigriventris* protractor electromotoneurons. In addition, the protractor motoneurons of *S. grandiops* had a higher rheobase, which could explain why tonal sounds were generally shorter and composed of fewer oscillations than the EDs of *S. nigriventris*. Unlike *S. nigriventris*, most synodontid species investigated so far (*S. nigrita*, *S. obesus*, *S. eupterus*, *S. marmorata*, *S. schall*, and *S. caudovitattus*) can produce ED bursts and (or exclusively, depending on the authors) EDs composed of a single pulse or a pair of pulses (Baron et al., 1994; Boyle et al., 2014; Hagedorn et al., 1990; Orlov et al., 2017; Orlov, Baron, & Golubtsov, 2015). Due to these differences, recordings of individual motor and premotor neurons during ESA activity are needed to fully understand how motoneurons contribute to ED generation (e.g., see Bass & Baker, 1990; Chagnaud & Bass, 2014; Chagnaud et al., 2012). A major limitation of this approach compared to the patch‐clamp experiment presented here is that so far, we have been unable to elicit fictive calls from anesthetized fish.

### 4.3 Concluding comments

The contribution of neural mechanisms to the evolution of novel communication channels and behaviors remain largely unexplored (but see Bass & Baker, 1997; Hoke, Adkins‐Regan, Bass, McCune, & Wolfner, 2019; Katz, 2016). Synodontid catfish are of great interest in this regard because two signaling mechanisms have evolved from the same neuromuscular system within a single genus. While the interspecific differences in motoneuron physiology and, in turn, EMGs reported here likely contribute to species differences in the pulse repetition rate of the resultant behavioral signals, more species need to be investigated to confirm this hypothesis. It may yet be that properties of the protractor muscle‐derived electrocytes contribute to the spectral features of individual pulses as they do in the myogenic electric organ of other teleosts (Bass, 1986; Zakon, Zwickl, Lu, & Hillis, 2008). From a broad comparative perspective, the similarities observed in peripheral and central characters with other sonic and weakly electric teleosts suggest similar selective pressures favoring the evolution of shared mechanisms controlling these signaling behaviors among distantly related taxa.

## Supporting information


**Table S1** Principal Component Analysis of the variables obtained from the Elastic Spring Apparatus.
**Table S2**. Comparisons between the Elastic Spring Apparatus of *Synodontis grandiops* and *S. nigriventris*.
**Table S3**. Comparisons between the protractor motoneuron nuclei of *Synodontis grandiops* and *S. nigriventris*.
**Table S4**. Comparison of the electrophysiological data recorded for *S. grandiops* and *S. nigriventris*.
**Table S5**. Principal Component Analysis of protractor motoneuron electrophysiological variables.

## Data Availability

The data that support the findings of this study are available from the corresponding author upon request.
