# Supplementary material for: Neuroanatomical and neurophysiological mechanisms of acoustic and weakly electric signaling in synodontid catfish
Source: J Comp Neurol. 2020 Apr 21;528(15):2602–19. doi: 10.1002/cne.24920 (PMC7496807; doi:10.1002/cne.24920)
Supplement: Supplementary file 1 — Table S1 Principal Component Analysis of the variables obtained from the Elastic Spring Apparatus. Table S2. Comparisons between the Elastic Spring Apparatus of Synodontis grandiops and S. nigriventris. Table S3. Comparisons between the protractor motoneuron nuclei of Synodontis grandiops and S. nigriventris. Table S4. Comparison of the electrophysiological data recorded for S. grandiops and S. nigriventris. Table S5. Principal Component Analysis of protractor motoneuron electrophysiological variables. [file CNE-528-2602-s001.docx]

**Supporting information**

**Table S1. Principal Component Analysis of the variables obtained from the Elastic Spring Apparatus.** Based on the Kaiser-Guttman rule the first two principal components were kept.

|  | PC 1 | PC 2 |
| --- | --- | --- |
| Eigenvalue | 4.63 | 1.53 |
| % variance | 66.08 | 21.84 |
| Variables |  |  |
| Plate Surface | 0.44 | -0.15 |
| Plate Thickness | -0.30 | -0.51 |
| Stem Length | -0.43 | 0.28 |
| Process Length | 0.43 | -0.09 |
| Muscle Mass | 0.41 | 0.11 |
| Muscle Midline | -0.05 | 0.77 |
| Muscle Thickness | 0.43 | 0.17 |
| Observations |  |  |
| *Synodontis grandiops 1* | 1.72 | -0.06 |
| *Synodontis grandiops 2* | 1.34 | -0.68 |
| *Synodontis grandiops 3* | 2.64 | 0.13 |
| *Synodontis grandiops 4* | 1.42 | -0.37 |
| *Synodontis grandiops 5* | 2.58 | -0.30 |
| *Synodontis nigriventris 1* | -1.19 | 0.16 |
| *Synodontis nigriventris 2* | -1.91 | 1.21 |
| *Synodontis nigriventris 3* | -1.14 | 2.69 |
| *Synodontis nigriventris 4* | -2.66 | -1.89 |
| *Synodontis nigriventris 5* | -2.80 | -0.89 |

**Table S2. Comparisons between the Elastic Spring Apparatus of *Synodontis grandiops* and *S. nigriventris.*** Alpha level (0.05) corrected using the Sequential Bonferroni Correction. Significant differences are highlighted in bold. The effect size was obtained using two different methods, ‘Mean Difference’ (not standardized) and ‘Hedges’g’ (standardized) described in the online application www.estimationstats.com. For each method, the table displays the effect size and the upper and lower bounds for 95% confidence interval. Hedges’g over 0.8 are considered as large effects. M-W U: Mann-Whitney U test. NV: impossible to calculate the Hedges’ g for these data.

| Variables | Tests | Results | P-values | Alphas (corrected) | Effect size  (Mean difference) | Effect size  (Hedges’*g*) |
| --- | --- | --- | --- | --- | --- | --- |
| Plate Surface | Student T | t8=6.71 | **0.0002** | 0.0071 | -1.11 [-1.42, -0.836] | -3.83 [-5.63, -2.84] |
| Stem Length | M-W U | Z=0 | **0.0079** | 0.0083 | 0.34 [0.29, 0.40] | 6.39 [4.22, 9.55] |
| Process Length | M-W U | Z=0 | **0.0079** | 0.01 | -0.14 [ -0.19, -0.11] | -3.96 [-5.61, -2.79] |
| Muscle Thickness | M-W U | Z=0 | **0.0357** | 0.017 | -0.22 [-0.28, -0.18] | -5.69 [-6.2, -5.11] |
| Muscle Mass | M-W U | Z=0 | 0.0357 | 0.0125 | -0.003 [-0.004, -0.002] | -2.76 [-3.74, -1.97] |
| Plate Thickness | M-W U | Z=9 | 0.5476 | / | 0.01 [-0.0003, 0.03] | 0.91 [-1.11, 2.46] |
| Midline length | Student T | Z=7 | 0.5556 | / | 0.28 [-0.31, 0.80] | NV |

**Table S3. Comparisons between the protractor motoneuron nuclei of *Synodontis grandiops* and *S. nigriventris.*** Alpha level (0.05) corrected using the Sequential Bonferroni Correction. Significant differences are highlighted in bold. The effect size was obtained using two different methods, ‘Mean Difference’ (not standardized) and ‘Hedges’g’ (standardized) described in the online application www.estimationstats.com. For each method, the table displays the effect size and the upper and lower bounds for 95% confidence interval. Hedges’g over 0.8 are considered as large effects. MN: motoneurons. M-W U: Mann-Whitney U test. NV: impossible to calculate the Hedges’ g for these data.

| Variables | Tests | Results | P-values | Alphas (corrected) | Effect size  (mean difference) | Effect size  (Hedges’ *g*) |
| --- | --- | --- | --- | --- | --- | --- |
| Protractor MN Count | M-W U | Z=0 | **0.0061** | 0.017 | -88.7 [-133, -50.7] | -1.72 [-2.51, -0.79] |
| Protractor MN Nucleus Length | M-W U | Z=6 | 0.1636 | 0.025 | -0.006 [-0.011, 0.001] | -1.13 [-3.22, 0.981] |
| Protractor MN Diameter | M-W U | Z=0 | 0.1000 | / | 0.011 [0.004, 0.019] | NV |

**Table S4. Comparison of the electrophysiological data recorded for *S. grandiops* and *S. nigriventris.*** The effect size was obtained using two different methods, ‘Mean Difference’ (not standardized) and ‘Hedges’g’ (standardized) described in the online application www.estimationstats.com. For each method, the table displays the effect size and the upper and lower bounds for 95% confidence interval. Hedges’g over 0.8 are considered as large effects. AP: action potential. ADP: afterdepolarization. AHP: afterhyperpolarization. AP number R+10%: number of action potential for a stimulus 10% over the rheobase. M-W U: Mann-Whitney U test. NV: impossible to calculate the Hedges’ g for these data.

| Variables | Tests | Results | P-values | Alphas (corrected) | Effect size  (mean difference) | Effect size  (Hedges’ *g*) |
| --- | --- | --- | --- | --- | --- | --- |
| AHP half-width | M-W U | Z=0 | **<0.0001** | 0.0038 | 0.43 [0.29, 0.62] | 2.08 [1.48, 2.71] |
| Rheobase | Student T | t24=7.72 | **<0.0001** | 0.0042 | -1250 [-1540, -957] | -2.94 [-3.85, -2.01] |
| Tau | Student T | t23=4.1 | **0.0004** | 0.0045 | 1.05 [0.63, 1.63] | 1.59 [0.847, 2.21] |
| Resistance | Student T | t23=3.54 | **0.0018** | 0.005 | 27.8 [14.3, 47.9] | 1.38 [0.688, 2.03] |
| AP latency | Student T | t21=3.56 | **0.0019** | 0.0056 | 0.67 [0.33, 1.06] | 1.43 [0.688, 2.4] |
| ADP amplitude | M-W U | Z=13.5 | **0.0024** | 0.0063 | -2.29 [-3.49, -1.34] | -1.54 [-2.26, -0.831] |
| AHP amplitude | Student T | t21=3.28 | **0.0036** | 0.0071 | -9.55 [-14.30, -3.41] | -1.32 [-2.59, -0.137] |
| AP half-width | Student T | t21=1.98 | 0.0616 | 0.0083 | -0.023 [-0.043, -0.0004]. | -0.795 [-1.72, 0.108] |
| Resting potential | Student T | t24=1.57 | 0.1298 | / | 2.13 [-0.61, 4.37] | 0.598 [-0.24, 1.44] |
| Capacitance | Student T | t24=0.81 | 0.4255 | / | -7e-12 [-2e-11, 8e-12] | -0.309 [-1.02, 0.507] |
| AP number R+10% | M-W U | Z=74.5 | 0.6430 | / | 7.74 [-9.17, 33.30] | 0.279 [-0.471, 1.06] |
| Latency | Student T | t21=0.44 | 0.6633 | / | 3.61 [-11.10, 17.60] | 0.179 [-0.587, 1.13] |
| AP amplitude | Student T | t19=0.1 | 0.9233 | / | -0.71 [-6.39, 6.53] | -0.0409 [-0.994, 0.84] |

**Table S5. Principal Component Analysis of protractor motoneuron electrophysiological variables.** Based on the Kaiser-Guttman rule the first four principal components were kept.

|  | PC 1 | PC 2 | PC 3 | PC 4 |
| --- | --- | --- | --- | --- |
| Eigenvalue | 5.26 | 1.66 | 1.54 | 1.37 |
| % variance | 40.43 | 12.80 | 11.87 | 10.55 |
| Variables |  |  |  |  |
| Time constant *Tau* | 0.35 | 0.05 | 0.21 | -0.15 |
| Capacitance | -0.11 | -0.09 | 0.57 | -0.37 |
| Resting potential | 0.18 | -0.45 | -0.15 | -0.31 |
| Resistance | 0.34 | 0.15 | -0.18 | 0.15 |
| Latency (long stimuli) | -0.04 | 0.24 | 0.52 | -0.30 |
| Rheobase | -0.40 | -0.11 | -0.04 | 0.10 |
| AP amplitude | -0.03 | -0.48 | 0.29 | 0.45 |
| Action Potential latency | 0.35 | 0.17 | 0.14 | 0.29 |
| Action Potential half-width | -0.30 | 0.35 | -0.13 | -0.16 |
| Afterhyperpolarization amplitude | -0.31 | 0.30 | -0.23 | -0.01 |
| Afterhyperpolarization half-width | 0.41 | 0.10 | -0.06 | -0.01 |
| Afterdepolarization amplitude | -0.25 | -0.34 | 0.02 | 0.14 |
| Number of AP at rheobase + 10% | -0.11 | 0.32 | 0.35 | 0.54 |
| Observations |  |  |  |  |
| *Synodontis grandiops 1* | -0.34 | 1.10 | -2.11 | -1.70 |
| *Synodontis grandiops 2* | 0.14 | -0.71 | -1.97 | 0.34 |
| *Synodontis grandiops 3* | -1.67 | 1.20 | 2.94 | -0.18 |
| *Synodontis grandiops 4* | -1.09 | -2.33 | 1.76 | -1.19 |
| *Synodontis grandiops 5* | -2.15 | 3.14 | -0.95 | -0.84 |
| *Synodontis grandiops 6* | -2.37 | 0.64 | -0.84 | 0.31 |
| *Synodontis grandiops 7* | -2.76 | -0.92 | -0.16 | 1.45 |
| *Synodontis grandiops 8* | -1.51 | -0.03 | 1.07 | -1.04 |
| *Synodontis grandiops 9* | -1.49 | -0.61 | -0.19 | 0.46 |
| *Synodontis grandiops 10* | -2.20 | -0.74 | 0.69 | 1.32 |
| *Synodontis grandiops 11* | 0.19 | -1.12 | 0.41 | -1.73 |
| *Synodontis grandiops 12* | -0.56 | -2.90 | -0.79 | 0.83 |
| *Synodontis grandiops 13* | -3.96 | 1.19 | 0.01 | 1.32 |
| *Synodontis grandiops 14* | -2.30 | -0.99 | -2.68 | -0.09 |
| *Synodontis nigriventris* 1 | 4.21 | 0.26 | -0.75 | 0.01 |
| *Synodontis nigriventris* 2 | 2.75 | 0.47 | -0.40 | 0.93 |
| *Synodontis nigriventris* 3 | 3.64 | 1.07 | -0.18 | 0.37 |
| *Synodontis nigriventris* 4 | 3.71 | 0.01 | 0.08 | 1.33 |
| *Synodontis nigriventris* 5 | 4.80 | -0.41 | 0.04 | -0.46 |
| *Synodontis nigriventris* 6 | -0.24 | 1.57 | -0.65 | -0.76 |
| *Synodontis nigriventris* 7 | 1.34 | -0.52 | -0.14 | 1.51 |
| *Synodontis nigriventris* 8 | 0.62 | 1.88 | 1.83 | 2.17 |
| *Synodontis nigriventris* 9 | 1.09 | -0.32 | 1.22 | -1.27 |
| *Synodontis nigriventris* 10 | 0.09 | 0.31 | 0.41 | -2.40 |
| *Synodontis nigriventris* 11 | 0.12 | -0.76 | 0.76 | -0.75 |
| *Synodontis nigriventris* 12 | -0.08 | -0.47 | 0.59 | 0.05 |
